# Birth weight in relation to maternal and neonatal biomarker concentration of perfluorooctane sulfonic acid: a meta-analysis and meta-regression from a systematic review

**DOI:** 10.1038/s41370-025-00798-8

**Published:** 2025-08-22

**Authors:** J. M. Wright, K. M. Rappazzo, H. Ru, A. L. Lee, M. W. Dzierlenga, T. F. Bateson, E. G. Radke

**Affiliations:** 1 US Environmental Protection Agency, Office of Research and Development, Center for Public Health and Environmental Assessment, Chemical and Pollutant Assessment Division, Cincinnati, OH USA; 2 US Environmental Protection Agency, Office of Research and Development, Center for Public Health and Environmental Assessment, Public Health and Environmental Systems Division, Research Triangle Park, NC USA; 3 US Environmental Protection Agency, Office of Research and Development, Center for Public Health and Environmental Assessment, Chemical and Pollutant Assessment Division, Research Triangle Park, NC USA; 4 US Environmental Protection Agency, Office of Research and Development, Center for Public Health and Environmental Assessment, Chemical and Pollutant Assessment Division, Washington, DC USA

**Keywords:** Epidemiology, Perfluorinated chemicals, Meta-analysis, Maternal and fetal exposure/health, Vulnerable populations

## Abstract

**Background:**

Perfluorooctane sulfonic acid (PFOS) is a legacy chemical, that while banned in some countries, is still found in various environmental media and in nearly all humans given its long half-life.

**Objective:**

We examined mean birth weight (BW) differences in relation to PFOS exposure biomarkers using systematic review methods.

**Methods:**

We fit a random effects model to obtain the overall pooled effect and for stratified analyses examining biomarker sample type and timing, study confidence, scaling factors, and country of study origin. We also conducted a meta-regression to assess the impact of gestational age and other factors on the overall pooled effect.

**Results:**

We found a 30-gram BW deficit (β = −30.3 g; 95%CI: −41.6, −18.9) with each ln-unit PFOS increase based on 53 studies identified in the systematic literature review. We detected BW deficits across all study confidence levels (β range: −27 to −37 g per ln-unit increase) with the largest deficit in the *medium* confidence grouping (β = −36.6 g; 95%CI: −56.3, −16.8). We did not see evidence of a gradient of BW deficits across biomarker sample timing (β range: −24 to −39 g per ln-unit increase), but the smallest deficit in our primary analyses was detected for the 18 early sample timing studies (β = −23.6 g; 95%CI: −38.7, −8.6). Robust deficits were also seen across various subgroups including by geographical region of study origin (e.g., Asian studies), more restrictive early biomarker sample collection, and post-partum samples (β range: −16.9 to −30.6 g). For meta-regression analyses, none of the investigated factors explained significant heterogeneity across studies.

**Impact:**

We detected a statistically significant BW deficit of 30 grams per each ln-unit PFOS increase across all 53 studies in our meta-analysis; results were comparable in magnitude across study confidence, sample timing, and other strata. Unlike previous meta-analyses based on fewer studies, our results suggest that pregnancy hemodynamics do not fully explain the overall association. Characterization of the potential risk of developmental effects related to PFOS and other legacy chemicals will have important risk assessment and risk management ramifications in the future.

## Introduction

Perfluorooctane sulfonic acid (PFOS) is a legacy per- and polyfluoroalkyl substance (PFAS) first manufactured by the 3M Company in 1949. PFOS has been used for a variety of products often as a water and oil repellent including usage in pesticides, fire suppressants, and consumer products such as soil and stain resistance clothing [1]. PFOS has been phased out in the US for over two decades, but industrial production and commercial use are ongoing in other countries. So, despite replacement PFAS, such as perfluorobutane sulfonic acid, being used in lieu of PFOS, this legacy chemical is still routinely found in nearly every human and environmental media worldwide.

PFOS has been shown to be associated with a variety of health effects in toxicological and epidemiological studies including, most recently, some developmental effects and cardiovascular health impacts such as increased serum total cholesterol [2]. While many epidemiological studies have demonstrated an association between PFOS exposure and decreased birth weight (BW) based on maternal and newborn PFOS exposure measures, understanding the magnitude of the decrease in BW is complicated due to challenges in combining epidemiological study results based on different approaches regarding data expressions (e.g., results based on categorical vs. continuous exposure data, or natural vs. log-scaled data), as well as variation within and between studies including biomarker sample collection type and timing differences. The potential impact of pregnancy hemodynamics (e.g., blood volume increases over gestation) which may alter biomarker concentrations has been examined in previous studies mostly by stratified analyses of different combinations of biomarker sampling timing. For example, Dzierlenga et al. [3] showed a small decrease in mean BW (β = −3.2 g; 95% confidence interval (CI): −5.1, −1.3 per ng/mL PFOS increase) among 19 studies examining maternal and newborn PFOS exposure measures. Larger BW deficits were seen among the studies with late (second trimester, third trimester, second and third trimester combined, or cord blood) biomarker sampling (β = −7.2 g; 95%CI: −10.9, −3.4) versus the early (prepregnancy, first trimester, or first and second trimester combined) pregnancy sampling group (β = −1.4 g; 95%CI: −2.3, −0.4). Although limited by a smaller sample size and the combination of maternal and neonatal samples, their data suggest that some of these differences may be due to pregnancy hemodynamics. Furthermore, their meta-regression results showed no change in BW at the intercept, which corresponds to biomarker sampling at the very beginning of pregnancy. The authors interpreted this as the result in a hypothetical cohort where sample collection occurs near conception and that is therefore not affected by any changes in plasma PFOS concentration that might occur even during early pregnancy. Thus, some uncertainty remains as to whether an association is present among epidemiological studies with early biomarker sampling anticipated to be less impacted by pregnancy hemodynamics. Better delineation of this uncertainty and the underlying relationship between PFAS and developmental effects has public health import as well as risk assessment and management implications.

In this systematic review, meta-analysis, and meta-regression, we examine the available epidemiological literature to assess the relationship between PFOS and changes in BW. We consider differences in exposure characterization such as biomarker sample type (maternal and neonatal) and timing (before or after birth early or during pregnancy), study confidence, country of study origin and other study characteristics to examine the impact of these factors on the overall association of PFOS and change in BW. This comprehensive literature review and meta-analysis includes rescaling and re-expression of results to allow for direct comparability of studies that report data on different scales, data that varied by transformation status, and data presented for continuous and categorical contrasts. Rescaling and re-expressions were performed to allow for the most expansive inclusion of studies possible while minimizing loss of data from non-comparability that would preclude pooling across studies.

## Methods

### Literature search

We used systematic review principles detailed in the ORD Staff Handbook for IRIS Assessments [4], US EPA’s systematic review protocol for PFAS [5], and PRISMA ([6]; see Supplementary Table 1). We relied on literature search results from a prior effort by the US EPA to examine health effects related to PFOS [7]. Search strings used in the literature search are detailed in Supplementary Table 2 in Appendix A with no language restrictions. The following databases were searched: Medline, Web of Science, Toxline, and Toxic Substances Control Act Test Submissions. This literature search includes publication dates from January 1, 2013 to April 18, 2024. The temporal scope of this search was restricted to studies published after 2012, since an EPA report titled “Health Effects Support Documents” for PFOS identified earlier literature [8]. To ensure that our search includes all earlier relevant BW studies, we also manually examined the reference lists from published PFOS birth weight meta-analyses (i.e., citation chaining). We have also been involved in other efforts to identify new studies before and after our latest literature search update including from other PFAS human health assessments [9].

From the epidemiological results of all health effects that met the population, exposure, comparator, and outcome (PECO) criteria (Supplementary Table 3), developmental outcome studies were identified and examined for BW results. Following SWIFT Review filtering to identify relevant studies (see supplemental materials), literature search results were imported into either DistillerSR (Evidence Partners; https://www.evidencepartners.com/products/distillersr-systematic-review-software) or SWIFT ActiveScreener (Sciome; https://www.sciome.com/swift-activescreener/) software and were screened against the PECO criteria by two independent reviewers that allowed a for conflict resolution process at two stages (i.e., title/abstract and full text phases) [7]. Studies that met the PECO criteria were tagged as having relevant human data, relevant animal data (in a mammalian model), or a physiologically based pharmacokinetic model. Studies that did not meet the PECO criteria as determined by title/abstract screening but did appear to include potentially important supporting information were categorized according to the type of information they provided.

We focused on human epidemiological studies for this evaluation. Following full text screening, we identified 72 publications examining BW measures in relation to PFOS exposure biomarkers. From that list, studies were included in our meta-analysis if they: (1) reported regression coefficients (i.e., “betas” (βs)) for the association between BW changes and PFOS in both sexes individually or combined; (2) reported 95% CIs or other measures of variance such as a standard error or a *p*-value that allowed for CI estimation; (3) measured concentrations in maternal blood before, during or after pregnancy, or infant umbilical cord or heel stick collected after pregnancy. Following exclusion of publications of overlapping analyses from the same study populations, there were 53 distinct units of observation in the primary meta-analysis from 54 publications and 50 observations were used in the meta-regression (see supplemental materials including Supplementary Table 4).

### Data pre-processing

We extracted the following items from each study: citation year, enrollment years, country where study was conducted, study design, sample size, biomonitoring matrix (plasma, serum, whole blood, dried blood spot), sample timing category (see Supplementary Table 5 for more detail), central measure (e.g., mean, median or midpoint, range) of timing of sample collection, central measure (e.g., mean, median, midpoint, range) of gestational age at birth, mean BW, standard deviation (SD) BW, mean BW z-score (if applicable), whether samples included term births only, percent of preterm births, exposure mean, exposure SD, exposure interquartile range (IQR), exposure percentiles at 5%, 10%, 25%, 50%, 75%, 90%, 95%, quantile ranges (if applicable, e.g., tertile bounds), z-score conversion of exposure (if applicable), exposure contrast and scale (e.g., ln, log_2_, log_10_, per SD, per IQR, original units), BW measures (mean BW differences (in grams) and standardized measures such as z-scores), β coefficient relating the BW endpoint to exposure metric, and confidence interval for β. We also extracted adjustment sets for specific covariates (Yes/No) examined in regression models, including maternal age, parity, prenatal care, body mass index (or height and weight), pregnancy weight gain, interpregnancy interval, race, parental education, socioeconomic status, marital status, maternal smoking, maternal ethanol intake, gestational age, sex, glomerular filtration rate, sampling timing, or other PFAS.

### Data synthesis

#### Digitization, re-scaling, and re-expression of results before quantitative synthesis

Some studies (e.g., refs. [10, 11]) did not report β’s and CIs including standardized BW data. Thus, we used WebPlotDigitizer version 4.6 [12] to estimate these values from their figures for inclusion in our analysis. To increase the ability to evaluate study consistency and aid the pooling of the data, we rescaled data to similar scaling. As part of this effort, several types of re-expression were used (Table 1) to convert results to a natural log (ln)-unit increase (equivalent to a 2.7-fold increase in PFOS). First, concentrations of PFOS in whole blood or dried blood spots were re-expressed as serum equivalents based on relevant data [13, 14]. As detailed in the Additional Supplemental Materials section and in prior publications [3, 15, 16], studies that reported results for categories of exposure rather than continuous exposure were re-expressed as if exposure had been represented continuously in their models. Third, for studies in which results had been expressed as difference in BW per log unit of exposure and the base of the log was not e, the results were re-expressed as per unit log_e_, using the change of base rule. For example, if a β coefficient was presented with the units of difference in g/log_10_(ng/mL), results would be re-expressed as g/log_e_(ng/mL) by dividing β by 2.30 (log_e_ (10) = 2.30). Fourth, for studies in which results had been reported with g per ng/mL or g per log_e_ (1+ng/mL), the results were re-expressed to g/log_e_(ng/mL).

**Table 1:** Original study and re-expressed results based on reported PFOS exposure distributions in 53 studies (from 54 publications) used in primary analysis.

	Exposure Summary	Exposure Distribution	Original β		Re-expressed β (g/ln(ng/mL))
Study	Mean/Q50/GM (Dispersion)	(µ, σ)	β (95%CI)	Unit	β (95%CI)
Apelberg [31]	5.0 (3.4–7.9 IQR ng/mL)	(1.61, 0.62)	−69.0 (−149, 10.0)	g/ln(ng/mL)	−69.0 (−149.0, 10.0)^a^
Ashley-Martin [51] Girls	4.6 (3.2–6.8 IQR ng/mL)	(1.53, 0.56)	94.3 (−76.3, 264.9)	g/log10(ng/mL)	41.0 (−33.1, 115.1)^a^
Ashley-Martin [51] Boys	4.6 (3.2–6.8 IQR ng/mL)	(1.53, 0.56)	−11.2 (−174.3, 152)	g/log10(ng/mL)	−4.8 (−75.7, 66)^a^
Bach [52]	8.3 (6–10.8 IQR ng/mL)	(2.12, 0.44)	−14.0 (−40.0, 11.0)	g/IQR (ng/mL)	−24.5 (−70, 19.2)
Bell [53]	1.7 (1.1–2.4 IQR ng/mL)	(0.54, 0.56)	−18.3 (−42.4, 5.8)	g/z ln(1+ng/mL)	−32.1 (−74.4, 10.1)^b^
Buck Louis [54]	5.1 (3.4–8.0 IQR ng/mL)	(1.64, 0.63)	−6.3 (−28.5, 15.8)	g/SD ln(1+ng/mL)	−10.0 (−44.8, 24.8)^b^
Cai [32]	2.9 (1.9–4.8 IQR ng/mL)	(1.07, 0.69)	−10.5 (−22.7, 3.8)^c^	z/ln(ng/mL) %change	−33.1 (−77.4, 11.2)^a^
Callan [22]	2.3 (1.4 SD ng/mL)	(0.68, 0.56)	−69.0 (−231, 94.0)	g/ln(ng/mL)	−69.0 (−231.0, 94.0)^a^
Cao [23]	1.0 (0.6–1.8 IQR ng/mL)	(0.01, 0.80)	−15.5 (−66.6, 35.6)^d^	g/ng/mL	−16.2 (−69.9, 37.4)
Chang [33]	2.2 (1.5–3.2 IQR ng/mL)	(0.78, 0.60)	−7.0 (−48.0, 34.0)	g/log2(ng/mL)	−10.1 (−69.2, 49.1)^a^
Chen [34]	5.9 (2.0 GSD ng/mL)	(1.78, 0.67)	−110.2 (−176, −44.5)	g/ln(ng/mL)	−110.2 (−176, −44.5)^a^
Chu [55]	7.2 (4.4–11.9 IQR ng/mL)	(1.97, 0.75)	−83.3 (−133.2, −33.4)	g/ln(ng/mL)	−83.3 (−133.2, −33.4)^a^
Darrow [56]	13.9 (9.5–19.7 IQR ng/mL)	(2.63, 0.54)	−49.0 (−90.0, −8.0)	g/ln(ng/mL)	−49.0 (−90.0, −8.0)^a^
de Cock [35]	1.6 (1.2–1.9 33–67QR ng/mL)	(0.47, 0.52)	140.2 (−26.2, 306.5)^d^	g/ng/mL	228.0 (−42.6, 498.7)
Eick [57]	1.9 (1.2–3.1 IQR ng/mL)	(0.66, 0.72)	3.8 (−27.0, 34.5)^d^	g/ng/mL	7.5 (−53.8, 68.8)
Espindola Santos [24]	2.0 (1.1–5.2 IQR ng/mL)	(0.71, 1.18)	0.1 (−0.4, 0.5)^d^	z/log10(ng/mL)	14.2 (−99.2, 127.6)^a^
Gao [25]	4.1 (3.1–5.2 33–67QR ng/mL)	(1.40, 0.58)	1.9 (−28.0, 31.8)^c^	g/ng/mL	7.9 (−116.5, 132.2)
Gardener [10]	3.9 (2.6–5.9 IQR ng/mL)	(1.36, 0.61)	132.7 (−147.0, 412.4)^c,d^	g/ng/mL	529.4 (−586.5, 1645.3)
Govarts [58]	2.6 (1.7–3.8 IQR ng/mL)	(0.97, 0.60)	10.8 (−72.4, 94.1)	g/IQRz ln(ng/mL)	13.5 (−90.0, 116.9)^a^
Gyllenhammar [36]	13 (7.4–19.0 IQR ng/mL)	(2.56, 0.70)	−39.5 (−97.1, 18.1)	g/ln(ng/mL)	−39.5 (−97.1, 18.1)^a^
Hamm [37]	7.4 (2.0 GSD ng/mL)	(2.00, 0.69)	31.3 (−43.3, 105.9)	g/ln(ng/mL)	31.3 (−43.3, 105.9)^a^
Hjermitslev [38]	9.0 (6.3–13 IQR ng/mL)	(2.20, 0.53)	−7.2 (−15.9, 1.4)	g/ng/mL	−66.4 (−145.9, 12.6)
Kashino [39]	3.4 (2.6–4.7 IQR ng/mL)	(1.22, 0.44)	−35.0 (−109.0, 39.0)	g/log10(ng/mL)	−15.2 (−47.3, 16.9)^a^
Kwon [40]	0.6 (0.3–1.1 IQR ng/mL)	(−0.45, 0.98)	−49.4 (−95.6, −3.3)	g/ln(ng/mL)	−49.4 (−95.6, −3.3)^a^
Lauritzen [59] North	11.3 (7.0 SD ng/mL)	(2.26, 0.57)	74.0 (−31.0, 178.0)	g/ln(ng/mL)	74.0 (−31.0, 178.0)^a^
Lauritzen [59] South	17.3 (7.5 SD ng/mL)	(2.77, 0.41)	−292.0 (−500.0, −84.0)	g/ln(ng/mL)	−292.0 (−500.0, −84.0)^a^
Lenters [41]	9.4 (1.6 2SD ln(ng/mL))	(2.24, 0.80)	−68.8 (−152.9, 15.2)	g/2SD ln(ng/mL)	−43.0 (−95.6, 9.5)^a^
Lind [60] Boys	8.1 (6.0–11.0 IQR ng/mL)	(2.09, 0.45)	−17.0 (−130.0, 97.0)	g/ln(ng/mL)	−17.0 (−130.0, 97.0)^a^
Lind [60] Girls	8.1 (6–11 IQR ng/mL)	(2.09, 0.45)	92.0 (−15.0, 199.0)	g/ln(ng/mL)	92.0 (−15.0, 199.0)^a^
Luo [61]	5.0 (3.3–7.6 IQR ng/mL)	(1.61, 0.62)	−93.3 (−157.9, −28.8)	g/ln(ng/mL)	−93.3 (−157.9, −28.8)^a^
Maisonet [42] Girls	19.6 (16.6–23 33–67QR ng/mL)	(2.98, 0.37)	−9.6 (−15.0, −4.1)^d^	g/ng/mL	−189.2 (−297.3, −81.0)
Manzano-Salgado [62]	6.1 (2.7 SD ng/mL)	(1.71, 0.43)	0.4 (−32.5, 33.4)	g/log2(ng/mL)	0.6 (−46.9, 48.1)^a^
Marks [77] Boys	13.8 (11–17.7 IQR ng/mL)	(2.62, 0.35)	−8.5 (−15.9, −1.1)	g/ng/mL	−118.2 (−221.5, −14.9)
Meng [43]	30.1 (22.9–39.0 IQR ng/mL)	(3.40, 0.39)	−25.2 (−55.3, 4.8)	g/log2(ng/mL)	−36.4 (−79.8, 6.9)^a^
Mwapasa [23]	1.4 (4.3 SD ng/mL)	(−0.84, 1.53)	−261 (−457, −64)	g/ln(ng/mL)	−261.0 (−457.0, −64.0)^a^
Peterson [44]	1.3 (1–1.9 IQR ng/mL)	(0.29, 0.48)	−37.2 (−123.9, 49.6)	g/ln(ng/mL)	−37.2 (−123.9, 49.6)^a^
Robledo [45] Boys	21.6 (0.6 SD ln(ng/mL))	(3.07, 0.57)	37.5 (−73.5, 148.5)	g/SD ln(ng/mL)	66.1 (−129.5, 261.6)^a^
Robledo [45] Girls	12.4 (0.6 SD ln(ng/mL))	(2.52, 0.55)	14.2 (−81.8, 110.2)	g/SD ln(ng/mL)	25.7 (−148.6, 200.1)^a^
Sagiv [63]	25.7 (18.9–34.9 IQR ng/mL)	(3.25, 0.45)	−17.9 (−40.9, 5.1)	g/IQR (ng/mL)	−29.1 (−66.5, 8.3)
Sevelsted [46]	6.2 (5–7.7 IQR ng/mL)	(1.83, 0.33)	0.0 (−0.1, 0.0)^c^	z/ng/mL	−139.5 (−244, −34.9)
Shen [64]	4.3 (2.7–7.2 IQR ng/mL)	(1.46, 0.73)	37 (−71, 145)	g/log10(ng/mL)	16.1 (−30.8, 63.0)^a^
Shi [27]	1 (0.6–1.6 IQR ng/mL)	(−0.03, 0.68)	160.5 (−11.9, 332.8)	g/log10(ng/mL)	69.7 (−5.1, 144.5)^a^
Shoaff [65]	14 (9.6–18 IQR ng/mL)	(2.64, 0.47)	−6.2 (−14.4, 1.9)	g/ng/mL	−88.4 (−204.2, 27.4)
Siwakoti [47]	7.1 (4.8–10 IQR ng/mL)	(1.97, 0.54)	0 (−0.1, 0.1)^c^	z/IQR ln(ng/mL)	1.4 (−15.7, 18.5)^a^
Starling [66]	2.4 (1.5–3.7 IQR ng/mL)	(0.88, 0.67)	−13.8 (−53.8, 26.3)	g/ln(ng/mL)	−13.8 (−53.8, 26.3)^a^
Valvi [67]	27.2 (23.1–33.1 IQR ng/mL)	(3.3, 0.27)	−81 (−173, 11)	g/log2(ng/mL)	−116.9 (−249.6, 15.9)^a^
Wang [48]	0.7 (0.4–1.2 IQR ng/mL)	(−0.43, 0.81)	−54.5 (−149.1, 40.1)	g/ln(ng/mL)	−54.5 (−149.1, 40.1)^a^
Wang [49]	1.9 (1.4–2.9 IQR ng/mL)	(0.66, 0.53)	0.1 (−0.1, 0.2)^c^	z/log10(ng/mL)	9.5 (−26.6, 45.6)^a^
Wang [68]	11.5 (9.2–14.9 IQR ng/mL)	(2.44, 0.36)	0 (−0.1, 0)^c^	z/log2(ng/mL)	−25.6 (−70.4, 19.2)^a^
Whitworth [69]	13 (10.3–16.6 IQR ng/mL)	(2.56, 0.35)	−3.5 (−9.3, 2.4)	g/ng/mL	−45.9 (−121.8, 31.4)
Wikström [70]	5.4 (4–7.6 IQR ng/mL)	(1.68, 0.48)	−46 (−88, −3)	g/ln(ng/mL)	−46.0 (−88.0, −3.0)^a^
Workman [28]	2.6 (1.8 SD ng/mL)	(0.76, 0.63)	−50 (−160.1, 60.1)	g/ln(ng/mL)	−50.0 (−160.1, 60.1)^a^
Xiao [71]	20.9 (6.2 GSD ng/mL)	(3.04, 1.82)	−0.5 (−0.9, −0.1)^c^	z/log2(ng/mL)	−328.9 (−594.8, −63)^a^
Xu [29]	5.7 (4.2 SD ng/mL)	(1.52, 0.66)	−417.3 (−742.1, −92.4)	g/log10(ng/mL)	−181.2 (−322.3, −40.1)^a^
Yao [11]	4.6 (3.2–5.9 IQR ng/mL)	(1.52, 0.45)	−32.3 (−116.2, 51.6)	g/ln(ng/mL)	−32.3 (−116.2, 51.6)^a^
Zhang [30]	NR	NR	23 (−58, 103)	g/ln(ng/mL)	23.0 (−58.0, 103.0)^a^
Zhang [72]	3.7 (2.5–5.2 IQR ng/mL)	(1.29, 0.55)	−161.4 (−286.3, −54.6)	g/log2(ng/mL)	−232.9 (−413.1, −78.8)^a^
Zheng [50]	38.2 (22.5–75.1 IQR ng/mL)	(3.64, 0.89)	−72.1 (−177.3, 33.1)	g/ln(ng/mL)	−72.1 (−177.3, 33.1)^a^

Q50: 50% percentile (median), µ: mean of log-normal distribution, σ: standard deviation of log-normal distribution, β: beta coefficient, 95%CI: confidence interval, 33–67QR: 33 percentile to 67 percentile range; *GM* geometric mean, *IQR* Interquartile range, *NR* not reported;

^a^Studies reported results per ln(ng/mL), log2(ng/mL) or log10(ng/mL) that can be mathematically rescaled to per ln(ng/mL).

^b^Studies reported results based on other types of log transformation that need to be re-expressed to g/ln(ng/mL), including Buck Louis et al. [54] which reported results per ln(1+ng/mL) and Bell et al. [53] which reported results based on per ln(1+ng/mL) and rescaled by the standard deviation of ln transformed chemicals.

^c^Studies that originally reported β of BW z-score.

^d^For studies in which the original analysis was based on categorical results, the original results were converted to continuous results (presented here g/ng/ml) as described in the supplemental information; for all other studies, the original β values as reported by study authors are shown.

#### Study evaluation-risk of bias in individual studies

Per the Office of Research and Development Staff Handbook for IRIS Assessments, U.S. EPA [4] has developed a method of evaluating the risk of bias and study sensitivity in individual studies. The epidemiology domains in the EPA study evaluation are study sensitivity, participant selection, confounding, selective reporting, exposure measurement, outcome ascertainment, and analysis. For each domain, the judgement can be either good, adequate, deficient, or critically deficient. Two investigators evaluated each study, and any disagreements were resolved by discussion and a third reviewer when necessary. Based on the ratings in all domains, the overall “study confidence” was determined (on an outcome-specific basis). As noted in the U.S. EPA PFAS protocol [5], the study confidence ratings of *high*, *medium*, *low*, or *uninformative* were based on the reviewer(s) judgments across the evaluation domains and considers the likely impact that noted deficiencies (inadequate reporting, risk of bias, low sensitivity) have on the study-level results.

#### Statistical analysis

We performed meta-analyses and estimated summary effect estimates for BW difference in grams in relation to PFOS exposures using the metafor package in R version 4.4.1 (R Core Team 2024). We did this for all available studies that could be re-scaled and re-expressed to allow for a large sample size for stratified analyses and better comparability between studies building on standard methods in previous meta-analyses [3, 17]. To evaluate stability of meta-effects, we also performed meta-analyses for studies stratified by a variety of factors including study confidence, sample type and timing, country of study origin, categorical and non-categorical reported effect estimates, log- or natural scale based reported effect estimates, and for those with and without any re-expression. The consistency of results for a given meta-analytic summary measure were characterized using Cochran’s *Q* test (p_Q_) and I^2^. Based on Cochrane’s guidelines [18], I^2^ values of percent of the variation in the pooled estimate due to between-study heterogeneity below <40% were considered low heterogeneity, while values from 40 to 69% were moderate, and values of 70% or more were considered high heterogeneity. To assess the possibility of publication bias, we used funnel plots, Egger’s regression test for symmetry, and a trim/fill analysis to identify and impute likely unpublished studies [18, 19].

#### Meta-regression

We examined potential contributors to any observed heterogeneity in the summary effect estimates using a random effects meta-regression analysis in the metafor package in R. We performed meta-regression for all included studies, and for non-transformed and non-re-expressed subsets. Potential modifiers were examined in univariable models, and in multivariable models with adjustment for estimated central tendency of sample timing (continuous, linear). Factors considered as potential modifiers were adjustment for maternal age, parity, body mass index (or height and weight), pregnancy weight gain, gestational age, glomerular filtration rate, sampling timing, or other PFAS; and the study population characteristics of median PFOS concentration, continent, restriction to term births only, PFOS biomarker matrix, and mean BW. Meta-regression was performed when there were at least 10 studies that could be included in an analysis of whether a covariate is important [18], and with sufficient variability within the variable (e.g., if fewer than 5 studies adjusted for sex, sex would not be examined via meta-regression); we also required an estimate of central tendency of sampling timing to be included in the meta-regression analysis.

#### Stratified analysis of sample timing

We used various approaches to estimate central tendency of sample timing for stratified analyses. Typical reported measures of centrality include medians, means, and midpoints. If these values were not reported, we calculated midpoints of ranges, midpoints of trimesters (for the minimum of Trimester 1, we used 6 weeks to account for later pregnancy detection; for the maximum of Trimester 3, we used average age at birth or, if not reported, 40 weeks and 6 days), weighted mean of means, weighted mean of midpoints, or weighted mean of medians. In cases where more than one measure of centrality is reported, we preferentially used medians over means to account for possible skewness. Measures of spread included range, standard deviation, and trimester. The following definitions were used: pre-conception = 0 days; Trimester 1 = 0 days to 13 weeks and 6 days; Trimester 2 = 14 weeks and 0 days to 27 weeks and 6 days; Trimester 3 = 28 weeks and 0 days to birth; when not provided by study authors, the gestational age at/after birth is assumed to be 40 weeks and 6 days old [20].

Using the estimated central tendency of sample timing and reported sampling ranges, we classified study sampling time periods as “early”, “late”, and post-partum for our primary analysis (Supplementary Table 5). We defined “early” pregnancy as preconception sampling or any combination of first trimester samples (e.g., first through third trimester) and “late” as exclusive maternal second trimester or later pregnancy sampling (e.g., exclusive second trimester, exclusive third trimester or a combination of the two). Post-partum is defined as maternal samples collected at or after birth or samples collected from the infant following birth. We also assessed the impact of collapsing the post-partum and late pregnancy sampled studies together similar to a binary approach used in earlier meta-analyses [3, 17].

#### Sensitivity and additional analyses

We examined stratified BW differences based on how data were originally reported (natural vs. log scale and categorical or continuous contrasts) and whether data were re-expressed. Because cord blood samples might differ considerably from maternal plasma or serum collected earlier during/before pregnancy, and since a previous meta-analysis reported a stronger inverse BW association from Asian studies [3, 21] compared to non-Asian studies, we also examined these strata. We have previously observed some differences in BW studies of PFHxS and PFNA even when restricted to the earliest sampled studies [15, 16]; therefore, we examined subgroups of sample timing to explore the potential impact of pregnancy hemodynamics. We conducted a sensitivity analysis excluding standardized BW studies where we converted z-scores to mean BW differences. Lastly, we examined the impact of excluding six studies with categorical data.

## Results

Fifty-three studies that met the aforementioned criteria were included in our meta-analysis (Supplementary Table 4). Nine studies were *low* confidence [22–30] and 20 were *medium* confidence [31–50]. Twenty-four studies were classified as *high* confidence [10, 11, 51–72]. A total of 14 studies were re-expressed for exposure comparisons. For example, 12 studies were re-expressed to log scale exposure from the natural scale (including six studies that required conversion from categorical to continuous) and two other studies were converted from log(X + 1) exposure to log exposure (Table 1).

**Fig. 1:**
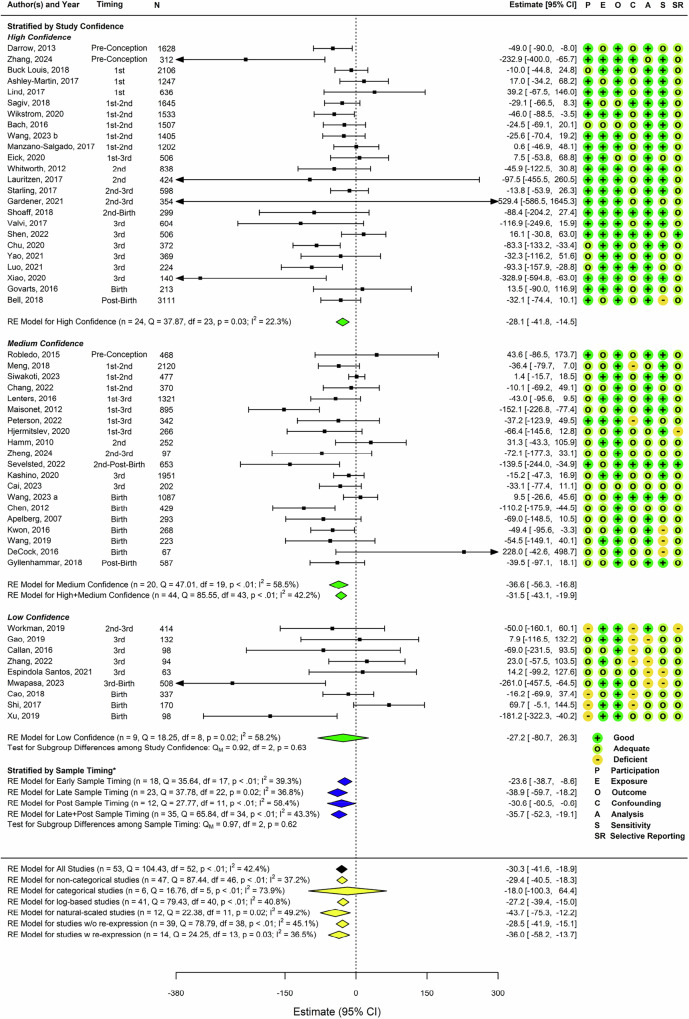
Forest plot and study evaluations of the 53 studies included in the meta-analysis on PFOS exposure and changes in birth weight. ^*^ Abbreviations: N: study sample size; n: number of studies; CI: Confidence Interval; 1st: Trimester 1; 2nd: Trimester 2; 3rd: Trimester 3; RE: random effect model; Q: Cochran’s Q test statistics; df: degree of freedom; p: *p*-value; I^2^: Higgin’s and Thompson’s I^2^; Q_M_: test statistics for subgroup differences. ^*^There are three sample timing strata; Early-pregnancy group: studies with biomarker samples taken either in the 1st trimester, a combination of 1st and 2nd trimester, or a combination of 1st, 2nd and 3rd trimester; Mid- to late-pregnancy group: measurements exclusively from the 2nd or 3rd trimester, a combination thereof, or a combination the 2nd, 3rd trimester, and at delivery; Post-pregnancy group: studies with biomarker samples from post-birth or at delivery/birth.

**Fig. 2:**
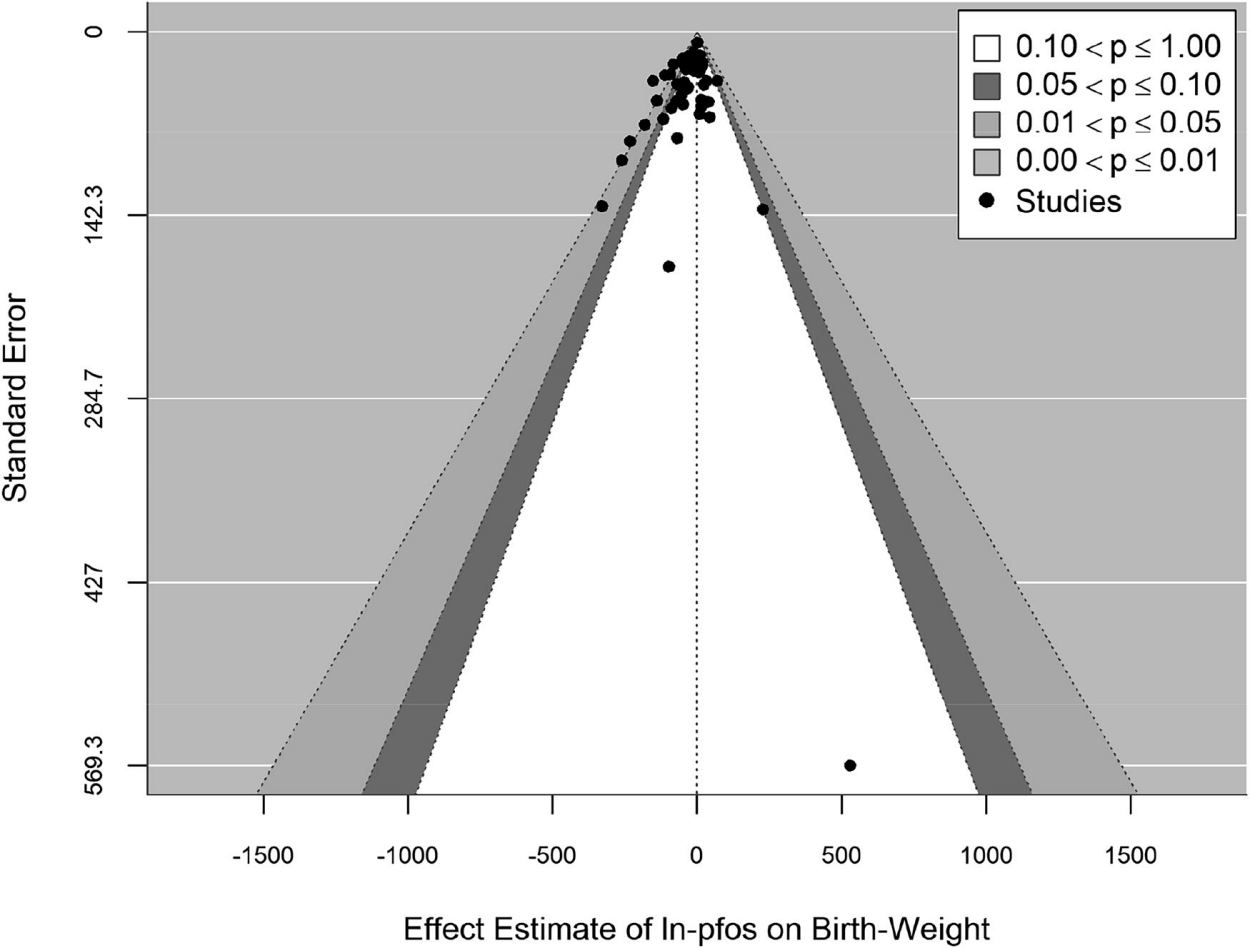
Enhanced Funnel Plot of 53 studies examining PFOS exposures and birth weight differences. *p*-value for the test for Funnel plot asymmetry *p* = 0.01.

Across all 53 studies, a 30-gram BW deficit (95%CI: −41.6, −18.9; I^2^ = 42.4%, *p*_Q_ < 0.0001) was seen with each increasing ln-unit PFOS increase (Fig. 1). All but one sub-group analysis had low to moderate between-study heterogeneity with I^2^ values ranging from 22% to 74%. There was some evidence of funnel plot asymmetry which might be indicative of potential publication bias (Fig. 2). The Egger’s test of symmetry identified that there may have been four unpublished studies showing generally smaller BW deficits. Imputation of four studies on the right side of the funnel plot decreased the overall effect estimates by 10% from −30 g to −27 g (β = −27.4 g; 95%CI: −39.1, -15.8; I^2^ = 44.1%, *p*_Q_ < 0.0001) (Supplementary Fig. 1).

Although no pattern was evident, we detected consistent BW deficits across all study confidence levels (β range: −27 to −37 g per ln-unit increase) (Fig. 1). The largest deficit was seen in the *medium* confidence grouping (β = −36.6 g; 95%CI: −56.3, −16.8) based on 20 studies. Deficits were similar in magnitude among the 24 *high* (β = −28.1 g; 95%CI: −41.8, −14.5) and 9 *low* confidence studies, with substantially more imprecision (β = −27.2 g; 95%CI: −80.7, 26.3) in the latter.

Our other stratified analyses showed differences between the 12 natural-scaled studies (β = −43.7 g; 95%CI: −75.3, −12.2 per ln-unit increase) and 41 log-based studies (β = −27.2 g; 95%CI: −39.4, −15.0 per ln-unit increase). The results for these 41 log-based studies were similar in magnitude and precision to 39 studies pooled together without re-expression (β = −28.5 g; 95%CI: −41.9, −15.1) as well as 14 studies that were re-expressed (β = −36.0 g; 95%CI: −58.2, −13.7). The results for the categorical studies that were re-expressed to each ln-unit increase were in the same direction but lower and more imprecise (β = −18.0 g; 95%CI: −100.3, 64.4). Exclusion of these six studies with categorical data (β = −29.4 g; 95%CI: −40.5, −18.3) had negligible impact on the overall results. The analysis of 16 Asian studies showed similar results (β = −30.2 g; 95%CI: −53.4, −6.9) to the entire database as well as the 37 non-Asian studies (β = −29.4 g; 95%CI: −42.1, −16.7) (Table 2). The result for the subset of 10 studies based on cord blood only (β = −29.6 g; 95%CI: −70.6, 11.3) was also similar in magnitude as was the exclusion of 4 individual studies for the ECHO Cohort pooled analysis results (β = −31.7 g; 95%CI: −44.2, −19.2) and 8 studies with z-score data (β = −32.5 g; 95%CI: −44.8, −20.2).

**Table 2:** Random effect estimates (β are in g/ln(ng/mL) of mean birth weight differences (and tests for heterogeneity) for PFOS exposures for sensitivity and additional analyses.

Set of Studies	*n*	β (g per ln(ng/mL))	95% Confidence Interval	I^2^ (%)	p_Q_
*Stratified Analyses*					
All studies	53	−30.3	−41.6, −18.9	42.4	<0.0001
Studies^a^ without re-expression of β	39	−28.5	−41.9, −15.1	45.1	0.0001
Studies^b^ with re-expression of β	14	−36.0	−58.2, −13.7	36.5	0.0289
Studies^c^ with log based β	41	−27.2	−39.4, −15.0	40.8	0.0002
Studies without log based β	12	−43.7	−75.3, −12.2	49.2	0.0216
Studies based on cord serum or plasma	10	−29.6	−70.6, 11.3	68.4	0.0012
Studies with maternal samples	43	−29.6	−41.0, −18.2	33.8	<0.0001
Studies from Asia	16	−30.2	−53.4, −6.9	61.5	0.0013
Studies outside of Asia	37	−29.4	−42.1, −16.7	29.4	0.0012
Earliest of the early subgroup (gestational age <=10 weeks)	9	−21.5	−42.8, −0.2	45.1	0.0126
Earliest of the early subgroup (gestational age <=13 weeks)	13	−16.9	−31.2, −2.6	25.3	0.0658
*Sensitivity Analyses*					
Four studies^d^ replaced by Padula et al. (2023)	50	−31.7	−44.2, −19.2	59.9	<0.0001
Excluding 8 studies with z-score data	45	−32.5	−44.8, −20.2	37.1	<0.0001
Excluding 6 studies with categorical data	47	−29.4	−40.5, −18.3	37.2	<0.0001

Symbols and abbreviations: *n* = number of studies, β = combined estimate of change in birth weight (g) per ln(ng/mL) PFOS exposure, I^2^ = % variation in the pooled effect due to study heterogeneity, p_Q_ = *p*-value for Cochran’s Q test for heterogeneity, T1 trimester 1.

^a^Studies reported results per ln(ng/mL), log_2_(ng/mL) or log_10_(ng/mL) that can be mathematically rescaled to per ln(ng/mL).

^b^Studies with re-expression of β: Bach et al. [52], Bell et al. [53], Buck Louis et al. [54], Cao et al. [23], de Cock et al. [35], Eick et al. [57], Gao et al. [25], Gardener et al. [10], Hjermitslev et al. [38], Maisonet et al. [42], Sagiv et al. [63], Sevelsted et al. [46], Shoaff et al. [65], Whitworth et al. [69].

^c^Studies reported results based on some types of log transformation, e.g., Buck Louis et al. [54] reported results per ln(1 + ng/mL) and Bell et al. [53] reported results based on per ln(1 + ng/mL) and rescaled by the standard deviation of ln transformed.

^d^Studies replaced by Padula et al. [75]: Chang et al. [33]; Eick et al. [57]; Sagiv et al. [63]; Starling et al. [66]; β of Padula et al. [75] was −12.8 g (95%CI: −30.6, 5.1) per. interquartile range ng/mL.

No examined variable explained heterogeneity in the meta-regression analyses, either in univariable models or in multivariable models adjusted for central tendency of sample timing (Supplementary Table 6). There was a small non-significant decrement in average change in BW across studies with increasing median PFOS level (β = −1.4 g; 95%CI: −2.9, 0.1) per unit increase). Coherent with the lack of influence of the sample timing central tendency estimate in the meta-regression, we also did not see evidence of a gradient of BW deficits across biomarker sample timing (β range: −24 to −39 g per ln-unit increase). Deficits were larger for the 23 late sample (β = −38.9 g; 95%CI: −59.7, −18.2) than the 12 post-partum sample studies (β = −30.6 g; 95%CI: −60.5, −0.6) and the 18 early sampled studies (β = −23.6 g; 95%CI: −38.7, −8.6). Results were not appreciably different (β = −21.5 g; 95%CI: −42.8, −0.2) based on the most restrictive earliest subset of nine studies with biomarker centrality measures ≤10 gestational weeks (Table 2), but BW deficits were slightly smaller when this window was extended to 13 studies with ≤13 gestational weeks (β = −16.9 g; 95%CI: −31.2, −2.6).

## Discussion

Our meta-analysis of 53 studies represents the largest systematic review of BW differences in relation to PFOS exposures published in the literature to date. Our literature review and extensive re-scaling and re-expression efforts allowed for inclusion of 25 additional non-duplicative studies than a 2020 meta-analysis [3] and 37 more than the 2022 meta-analysis by Gui et al. [21]. These meta-analyses were performed with different scales of exposure and are therefore not directly comparable. The association (β = −35 g) reported per ln ng/mL increase by Gui et al. [21] was more than an order of magnitude larger than the association (−3 g) reported by Dzierlenga et al. [3] per ng/mL increase in the natural scale. Our overall pooled results (β = −30.3 g; 95%CI: −41.6, −18.9) are in alignment with Gui et al. [21] despite differences in analytical approach and their considerably fewer number of included studies. Given the uncertainty related to re-expression of log transformed data [73, 74], we stratified results by originally reported exposure scaling. We found a larger mean BW reduction in 12 natural-scaled studies that were re-expressed to the log-scale (β = −44 g per ln g ng/mL) than the 41 log-based studies (β = −27 g per ln ng/mL). While these previous studies have characterized uncertainty in the application of re-expression methods from exposure on the log scale to the natural scale, no analogous work has examined re-expression from the natural scale to the log scale. This makes it difficult to determine what degree the re-expression itself plays in the discrepancy between studies originally reported in the natural scale compared to the log scale. Further research is needed to evaluate the uncertainty resulting from re-expression from the natural scale to the log scale. The type of systematic review presented here would benefit from future epidemiological studies reporting results in multiple scales.

A strength of our analyses was the examination of study sensitivity and the risk of bias within individual studies. Multiple reviewers evaluated the study quality and study sensitivity which allowed for these explanatory factors to also be evaluated in our analyses. We did not find a pattern in BW deficits as results were robust across all deficits across all study confidence levels (β range: −27 to −37 g per ln-unit increase). Other sub-group analyses including the 16 Asian studies (β = −30.2 g; 95%CI: −53.4, −6.9) showed a deficit similar in magnitude to 37 non-Asian studies (β = −29.4 g; 95%CI: −42.1, −16.7) and our overall meta-analysis of 53 studies (β = −30.3 g; 95%CI: −41.6, −18.9).

We analyzed additional biomarker sample categories according to the type and timing of sample collection to evaluate potential stratified differences in post-partum sub-groups as well as samples collected during pregnancy that may be due to the potential impact of pregnancy hemodynamics. Per each ln-unit increase, deficits were slightly larger in the 23 late-pregnancy (β = −38.9 g; 95%CI: −59.7, −18.2) than 12 post-partum sampled studies (β = −30.6 g; 95%CI: −60.5, −0.6) and 10 umbilical cord studies (β = −29.6 g; 95%CI: −70.6, 11.3). Although there were minimal differences between the latter two post-partum categorical groups, the 10 umbilical cord sampled studies represent the most homogeneous post-partum subset. Our primary analysis of 18 early sample timing studies (β = −23.6 g; 95%CI: −38.7, −8.6) was comparable in magnitude to an even more restrictive subset of 9 studies with the earliest sampling (β = −21.5 g; 95%CI: −42.8, −0.2) based on sampling measures <= 10 gestational weeks. These results suggest that observed deficits for PFOS are not entirely attributable to pregnancy hemodynamics which was consistent with our meta-regression results. We acknowledge some uncertainty in the pregnancy window grouping approaches for the meta-analysis and for the continuous measures used in the meta-regression especially given that many studies are utilizing biomarker samples that crossed different sensitive time periods and because our analysis of those data are based on the assumption of linearity. Thus, we relied on measures of centrality estimates to bin studies into post-partum measures, as well as early- and late-pregnancy windows, since few studies collected samples within a specific trimester alone. Although we did not see major differences by sampling timing beyond generally smaller deficits in earlier sampled studies, future studies with serial sampling and/or those that have less inter-individual sampling variability and earlier trimester one samples may better inform on any potential impact of pregnancy hemodynamics. Additional research is also needed to better understand transplacental transfer of PFOS and to enable more analyses of homogeneous subgroups that may better delineate the potential impact of hemodynamics from other determinants.

We conducted several sensitivity analyses to examine how robust our results were to various analytical approaches and assumptions and study result inclusion. For example, a multi-study pooled analysis of various ECHO birth cohorts by Padula et al. [75] was not included in our primary analysis but was considered as part of a sensitivity analysis. Inclusion of various individual ECHO cohort studies in the main analysis afforded us an additional strength in that each of the publications [33, 57, 63, 66] reported their unique biomarker sample timing estimates which allowed for subgroup meta-analyses and inclusion in our meta-regression. Given the disparate sampling times across ECHO Cohorts, Padula et al. [75] was not included in the meta-regression in lieu of the original ECHO cohort studies. Our sensitivity analysis replacing those four publications with the overall result from Padula et al. [75] did not impact the overall pooled effect estimate (e.g., β = −30.3 g vs. −31.7 g).

We observed low to moderate between-study heterogeneity across every sub-group in this review except for one, along with some evidence of potential publication bias. The lone sub-group that showed high heterogeneity (I^2^ = 73.9%) was based on six categorical studies that showed BW deficits consistent in magnitude with other analyses. These studies were originally conducted based on a categorical analysis and were converted to an approximate continuous effect using methods applied in our previous meta-analyses [3, 15, 16]. While this method has the substantial benefit of allowing additional observations to be included in the data integration, it does introduce some uncertainty. Evaluation of this method using a convenience sample of studies within this meta-analysis that reported continuous and categorical analyses resulted in an approximated continuous effect estimate that was substantially different from the reported analyses in two out of the five cases examined (Supplementary Table 7). Some of the required assumptions of the method are that exposure is lognormally distributed and that the dose-response relationship is linear. In cases where these assumptions are not appropriate, the method will be less accurate. Furthermore, the effect of influential observations, both within a single exposure category and between different categories of exposure, cannot be captured as the re-expression necessarily relies on summary statistics reported by the authors. While we acknowledge that more evaluation of this method is needed, inclusion of these data re-expressed as continuous results had minimal impact on the overall pooled analyses or observed heterogeneity across studies. When possible, epidemiological studies should present results based on both continuous and categorical expression in published manuscripts to allow for data integration without requiring re-expression methods that approximate results in a similar scale.

We acknowledge that some error may result from our conversion of standardized BW differences by multiplying the z-score by the overall population BW SD. Given the lack of stratified BW data for various factors, such as sex and gestational age, we recognize some uncertainty may exist especially among those studies based on external standardization approaches. However, results were generally robust in sensitivity analyses after exclusion of eight studies with z-score data converted to mean BW differences. Epidemiological studies that report results for both standardized and non-standardized results may aid quantification of this uncertainty. Future systematic reviews would also benefit from epidemiological studies that provide more detailed information regarding standardized BW calculations that would allow secondary users to back-calculate mean BW differences especially when based on external standardization.

While no examined factors explained observed heterogeneity, the general stability of the meta-effect estimates in magnitude and direction across examined strata is reassuring. Exploration of other potential sources of heterogeneity might delve into more specific population or study characteristics, beyond exposure characterization, country of study origin, exposure levels and contrasts, or adjustment factors. In future research, harmonization of exposure characterization and expression could reduce uncertainty and allow for improved assessment of the overall association between PFAS and BW. While it did not explain any observed heterogeneity, we did see a small nonsignificant decrement in average change in BW with increased median PFOS. This met our expectation that the response to PFOS may be non-linear across exposure ranges, as well as acknowledging that the ln-unit change at higher exposure levels is much larger than that at lower exposure levels. It is possible that further exploration of exposure-response curves could provide additional context and explain some of the heterogeneity across studies as highlighted recently by others [76].

## Conclusions

Overall, we detected a 30-gram BW deficit in our pooled analysis as well as deficits of at least 17 grams per each ln-unit increase of PFOS across every stratified analysis and sensitivity analysis. Our comprehensive literature search and systematic approach and re-expression techniques allowed a considerably larger number of studies to be examined than previous publications and enabled various stratified analyses to examine between study-heterogeneity. Some uncertainty remains as to any quantitative impact of pregnancy hemodynamics on this literature base, so epidemiological studies with more homogeneous sampling strategies and/or repeated measurements during pregnancy may be able to further inform this methodological issue. These types of meta-analytical data that can address important sources of uncertainty and variability are likely to be increasingly used in future risk assessments that derive dose-response functions for developmental effects and/or help inform cost-benefit analyses and risk management interventions.

## Supplementary information

Supplemental Materials
