# Supplementary material for: Birth weight in relation to maternal and neonatal biomarker concentration of perfluorooctane sulfonic acid: a meta-analysis and meta-regression from a systematic review
Source: J Expo Sci Environ Epidemiol. 2025 Aug 22;35(6):1030–40. doi: 10.1038/s41370-025-00798-8 (PMC12583133; doi:10.1038/s41370-025-00798-8)
Supplement: Supplementary file 1 — Supplemental Materials [file 41370_2025_798_MOESM1_ESM.docx]

*Birth weight in relation to maternal and neonatal biomarker concentration of perfluorooctane sulfonic acid: a meta-analysis and meta-regression from a systematic review*

Wright JM, Rappazzo KM, Ru H, Lee AL, Dzierlenga MW, Bateson TF, Radke EG.

*Journal of Exposure Science & Environmental Epidemiology*; https://doi.org/10.1038/s41370-025-00798-8

**Supplemental Materials**

Contents

[Supplemental Table 1. PRISMA Checklist 2](#_Toc192598264)

[Supplemental Table 2. Literature Search Algorithm 4](#_Toc192598265)

[Literature Search Screening 5](#_Toc192598266)

[Supplemental Table 3. Populations, Exposure, Comparator, and Outcome (PECO) Criteria and Study Eligibility Criteria. 6](#_Toc192598267)

[Study Inclusion and Exclusion 6](#_Toc192598268)

[Supplemental Table 4. Study Characteristics of 53 informative PFOS studies included from 54 publications. 8](#_Toc192598269)

[Supplemental Table 5. Central Tendency Sample Timing Estimation Procedures and Notes. 12](#_Toc192598270)

[Supplemental Table 6: Results from meta-regression analyses for 50 studies 16](#_Toc192598271)

[Additional Supplemental Materials 20](#_Toc192598272)

[Conversion of Categorical Exposure Results to Continuous 20](#_Toc192598273)

[Supplemental Table 7. Comparison of continuous effect estimates (and confidence intervals) that were reported with values estimated from categorical analyses reported by the authors. β values are in units of g/ng/mL unless otherwise noted. 21](#_Toc192598274)

[Supplemental Figure 1. Enhanced Funnel Plot of 53 studies included, plus 4 studies imputed, examining PFOS exposures and birth weight differences. 22](#_Toc192598275)

References………………………………………………………………………………………………………………………………………………………………………………………………………......23

Supplemental Table 1. PRISMA Checklist (see also Moher et al., 2010)

| **Section and Topic** | **Item #** | **Checklist item** | **Location where item is reported** |
| --- | --- | --- | --- |
| **TITLE** | | |  |
| Title | 1 | Identify the report as a literature review. | Page 1 lines 1-2 |
| **ABSTRACT** | | |  |
| Abstract | 2 | Provide a structured summary including, as applicable: background; objectives; data sources; study eligibility criteria, participants, and interventions; study appraisal and synthesis methods; results; limitations; conclusions and implications of key findings.  See the [PRISMA 2020 for Abstracts checklist](http://www.prisma-statement.org/Extensions/Abstracts.aspx) for the complete list. | Page 1 lines 28-46 |
| **INTRODUCTION** | | |  |
| Rationale | 3 | Describe the rationale for the review in the context of existing knowledge, i.e., what is already known about your topic. | Pages 2-3 lines 68-92, |
| Objectives | 4 | Provide an explicit statement of the objective(s) or question(s) the review addresses with reference to participants, interventions, comparisons, outcomes, and study design (PICOS). | Pages 3-4 lines 39-102 |
| **METHODS** | | |  |
| Eligibility criteria | 5 | Specify the inclusion and exclusion criteria for the review and how studies were grouped for the syntheses with study characteristics (e.g., PICOS, length of follow-up) and report characteristics (e.g., years considered, language, publication status) used as criteria for eligibility, giving rationale. | Page 5 lines 131-140  Suppl Pages 6-7 lines 63-110 |
| Information sources | 6 | Specify all databases, registers, websites, organisations, reference lists and other sources searched or consulted to identify studies. Specify the date when each source was last searched or consulted. | Page 4 lines 108-118 |
| Search strategy | 7 | Present the full search strategies for all databases, registers and websites, including any filters and limits used. | Suppl Pages 4-5 |
| Selection process | 8 | State the process for selecting studies (i.e., screening, eligibility).  Specify the methods used to decide whether a study met the inclusion criteria of the review, including how many reviewers screened each record and each report retrieved, whether they worked independently, and if applicable, details of automation tools used in the process. | Page 4 lines 119-130 |
| Study risk of bias assessment | 11 | Specify the methods used to assess risk of bias in the included studies, including details of the tool(s) used, how many reviewers assessed each study and whether they worked independently, and if applicable, details of automation tools used in the process. | Page 7 lines 181-192 |
| **RESULTS** | | |  |
| Study selection | 16a | Describe the results of the search and selection process, from the number of records identified in the search to the number of studies included in the review, ideally using a flow diagram. | Page 10 lines 262-263  Suppl Pages 6-7 lines 63-110 |
|  | 16b | Cite studies that might appear to meet the inclusion criteria, but which were excluded, and explain why they were excluded. | Suppl Pages 6-7 lines 63-110 |
| Study characteristics | 17 | Cite each included study and present its characteristics (e.g., study size, PICOS, follow-up period). | Suppl Pages 8-11 |
| Risk of bias in studies | 18 | Present assessments of risk of bias for each included study. | Figure 1 |
| Results of individual studies | 19 | For all outcomes, present, for each study: (a) summary statistics for each group (where appropriate) and (b) an effect estimate and its precision (e.g. confidence/credible interval), ideally using structured tables or plots. | Table 1 |
| **DISCUSSION** | | |  |
| Discussion | 23a | Provide a general interpretation of the results in the context of other evidence. | Page 13-142 lines 319-338 |
|  | 23b | Discuss any limitations of the evidence included in the review. | Page 14 lines 357-360,  Page 16 lines 385-395,  Page 16 lines 400-403 |
|  | 23c | Discuss any limitations of the review processes used. | Pages 7-8 lines 73-98 |
|  | 23d | Discuss implications of the results for practice, policy, and future research. | Pages 17-18 lines 426-434 |
| **OTHER INFORMATION** | | |  |
| Registration and protocol | 24a | Provide registration information for the review, including register name and registration number, or state that the review was not registered. | N/A |
|  | 24b | Indicate where the review protocol can be accessed, or state that a protocol was not prepared. | PROSPERO |
|  | 24c | Describe and explain any amendments to information provided at registration or in the protocol. | N/A |
| Support | 25 | Describe sources of financial or non-financial support for the review, and the role of the funders or sponsors in the review. | N/A |
| Competing interests | 26 | Declare any competing interests of review authors. | N/A |
| Availability of data, code, and other materials | 27 | Report which of the following are publicly available and where they can be found: template data collection forms; data extracted from included studies; data used for all analyses; analytic code; any other materials used in the review. | N/A |

# Supplemental Table 2. Literature Search Algorithm

| Web of Science | ((TS="perfluorooctanoic acid" OR TS="perfluorooctane sulfonic acid") AND PY=(2013-2019) OR (TS="2,2,3,3,4,4,5,5,6,6,7,7,8,8,8-pentadecafluoro-Octanoic acid" OR TS="2,2,3,3,4,4,5,5,6,6,7,7,8,8,8-pentadecafluorooctanoic acid" OR TS="3,3,4,4,5,5,6,6,6-nonafluoro-2-oxo-Hexanoyl fluoride" OR TS="3,3,4,4,5,5,6,6,6-nonafluoro-2-oxohexanoyl fluoride" OR TS="Hexanoyl fluoride, 3,3,4,4,5,5,6,6,6-nonafluoro-2-oxo-" OR TS="Octanoic acid, 2,2,3,3,4,4,5,5,6,6,7,7,8,8,8-pentadecafluoro-" OR TS="Pentadecafluoro-1-octanoic acid" OR TS="Pentadecafluoro-n-octanoic acid" OR TS="Pentadecafluorooctanoic acid" OR TS="Perfluorocaprylic acid" OR TS="Perfluoroctanoic acid" OR TS="Perfluoroheptanecarboxylic acid" OR TS="perfluorooctanyl sulfonate" OR TS="Perfluorooctanoic acid" OR TS="Octanoic acid, pentadecafluoro-" OR TS="Perfluorooctanoate" OR TS="perfluorooctane sulfonate" OR TS="A 5717" OR TS="EF 201" OR TS="Eftop EF 201" OR TS="Perfluoro-1-heptanecarboxylic acid" OR TS="1,1,2,2,3,3,4,4,5,5,6,6,7,7,8,8,8-Heptadecafluoro-1-octanesulfonic acid" OR TS="1-Octanesulfonic acid, 1,1,2,2,3,3,4,4,5,5,6,6,7,7,8,8,8-heptadecafluoro-" OR TS="1-Perfluorooctanesulfonic acid" OR TS="EF 101" OR TS="Eftop EF 101" OR TS="Heptadecafluoro-1-octanesulfonic acid" OR TS="Heptadecafluorooctane-1-sulphonic acid" OR TS="Perfluorooctane sulfonate" OR TS="perfluorooctane sulfonate" OR TS="Perfluorooctane sulfonic acid" OR TS="Perfluorooctanesulfonic acid" OR TS="Perfluorooctylsulfonic acid" OR TS="perfluorooctane sulphonate" OR TS="perfluorooctane sulfonate" OR TS="1-Octanesulfonic acid, heptadecafluoro-"OR TS="Heptadecafluorooctanesulfonic acid" OR TS="Perfluoro-n-octanesulfonic acid" OR TS="Perfluorooctane Sulphonic Acid" OR TS="Perfluorooctanesulfonate" OR TS="Perfluorooctylsulfonate" OR ((TS="PFOA" OR TS="PFOS") AND (TS="fluorocarbon*" OR TS="fluorotelomer*" OR TS="polyfluoro*" OR TS="perfluoro-*" OR TS="perfluoroa*" OR TS="perfluorob*" OR TS="perfluoroc*" OR TS="perfluorod*" OR TS="perfluoroe*" OR TS="perfluoroh*" OR TS="perfluoron*" OR TS="perfluoroo*" OR TS="perfluorop*" OR TS="perfluoros*" OR TS= "perfluorou*" OR TS="perfluorinated" OR TS="fluorinated" OR TS="PFAS"))) AND PY=(2013-2019)) |
| --- | --- |
| PubMed | (335-67-1[rn] OR 1763-23-1[rn] OR 45298-90-6[rn] OR "perfluorooctanoic acid"[nm] OR "perfluorooctane sulfonic acid"[nm]) AND (2013/01/01:3000[pdat] OR 2013/01/01:3000[mhda] OR 2013/01/01:3000[edat] OR 2013/01/01:3000[crdt]) OR (("2,2,3,3,4,4,5,5,6,6,7,7,8,8,8-pentadecafluoro-Octanoic acid"[tw] OR "2,2,3,3,4,4,5,5,6,6,7,7,8,8,8-pentadecafluorooctanoic acid"[tw] OR "3,3,4,4,5,5,6,6,6-nonafluoro-2-oxo-Hexanoyl fluoride"[tw] OR "3,3,4,4,5,5,6,6,6-nonafluoro-2-oxohexanoyl fluoride"[tw] OR "Hexanoyl fluoride, 3,3,4,4,5,5,6,6,6-nonafluoro-2-oxo-"[tw] OR "Octanoic acid, 2,2,3,3,4,4,5,5,6,6,7,7,8,8,8-pentadecafluoro-"[tw] OR "Pentadecafluoro-1-octanoic acid"[tw] OR "Pentadecafluoro-n-octanoic acid"[tw] OR "Pentadecafluorooctanoic acid"[tw] OR "Perfluorocaprylic acid"[tw] OR "Perfluoroctanoic acid"[tw] OR "Perfluoroheptanecarboxylic acid"[tw] OR “perfluorooctanyl sulfonate”[tw] OR "Perfluorooctanoic acid"[tw] OR "Octanoic acid, pentadecafluoro-"[tw] OR "Perfluorooctanoate"[tw] OR “perfluorooctane sulfonate”[tw] OR "A 5717"[tw] OR "EF 201"[tw] OR "Eftop EF 201"[tw] OR "Perfluoro-1-heptanecarboxylic acid"[tw] OR "1,1,2,2,3,3,4,4,5,5,6,6,7,7,8,8,8-Heptadecafluoro-1-octanesulfonic acid"[tw] OR "1-Octanesulfonic acid, 1,1,2,2,3,3,4,4,5,5,6,6,7,7,8,8,8-heptadecafluoro-"[tw] OR "1-Perfluorooctanesulfonic acid"[tw] OR "EF 101"[tw] OR "Eftop EF 101"[tw] OR "Heptadecafluoro-1-octanesulfonic acid"[tw] OR "Heptadecafluorooctane-1-sulphonic acid"[tw] OR "Perfluorooctane sulfonate"[tw] OR "perfluorooctane sulfonate"[tw] OR "Perfluorooctane sulfonic acid"[tw] OR "Perfluorooctanesulfonic acid"[tw] OR "Perfluorooctylsulfonic acid"[tw] OR "perfluorooctane sulphonate" [tw] OR “perfluorooctane sulfonate”[tw] OR "1-Octanesulfonic acid, heptadecafluoro-"[tw] OR "Heptadecafluorooctanesulfonic acid"[tw] OR "Perfluoro-n-octanesulfonic acid"[tw] OR "Perfluorooctane Sulphonic Acid"[tw] OR "Perfluorooctanesulfonate"[tw] OR "Perfluorooctylsulfonate"[tw] OR (("PFOA"[tw] OR "PFOS"[tw]) AND (fluorocarbon*[tw] OR fluorotelomer*[tw] OR polyfluoro*[tw] OR perfluoro-*[tw] OR perfluoroa*[tw] OR perfluorob*[tw] OR perfluoroc*[tw] OR perfluorod*[tw] OR perfluoroe*[tw] OR perfluoroh*[tw] OR perfluoron*[tw] OR perfluoroo*[tw] OR perfluorop*[tw] OR perfluoros*[tw] OR perfluorou*[tw] OR perfluorinated[tw] OR fluorinated[tw] OR PFAS[tw]))) AND (2013/01/01:3000[pdat] OR 2013/01/01:3000[mhda] OR 2013/01/01:3000[edat] OR 2013/01/01:3000[crdt])) |
| Toxline | @AND+@OR+("perfluorooctane sulfonate"+"pfos"+"perfluorooctanesulfonic acid"+"perfluorooctane sulfonic acid"+"perfluorooctane sulphonate"+"perfluorooctane sulfonate"+"perfluorooctanyl sulfonate"+"Heptadecafluorooctane-1-sulphonic"+"Heptadecafluoro-1-octanesulfonic acid"+"1,1,2,2,3,3,4,4,5,5,6,6,7,7,8,8,8-heptadecafluoro-1-octanesulfonic acid"+"perfluorooctanoate"+"perfluorooctanoic acid"+"perfluoroctanoic acid"+"pfoa"+"2,2,3,3,4,4,5,5,6,6,7,7,8,8,8-pentadecafluorooctanoic acid"+"Pentadecafluoro-1-octanoic acid"+"Pentadecafluoro-n-octanoic acid"+"Octanoic acid, pentadecafluoro-"+"Perfluorocaprylic acid"+"Pentadecafluorooctanoic acid"+"perfluoroheptanecarboxylic acid"+@TERM+@rn+335-67-1+@TERM+@rn+1763-23-1+@TERM+@rn+45298-90-6)+@NOT+@org+pubmed+@AND+@RANGE+yr+2013+2019 |
| TSCATS | @AND+@OR+@rn+”335-67-1”+@AND+@org+TSCATS+@NOT+@org+pubmed  @AND+@OR+@rn+"1763-23-1"+@AND+@org+TSCATS+@NOT+@org+pubmed |

Abbreviation: TSCATS: Toxic Substances Control Act Test Submissions

## Literature Search Screening

After deduplication (i.e., removal of duplicate results) in HERO, the database search results were imported into SWIFT Review software for filtering/prioritization. SWIFT Review identifies those references most likely to be applicable to human health risk assessment (https://www.sciome.com/swift-review/; see also Howard et al. [77]). In brief, SWIFT Review has preset literature search strategies (“filters”) developed and applied by information specialists to identify and prioritize studies that are most likely to be useful for identifying human health content from those that likely are not (e.g., studies on analytical methods). The filters function like a typical search strategy in which studies are tagged as belonging to a certain category if the terms in the filter literature search strategy appear in title, abstract, keyword, and/or medical subject headings fields content. The applied SWIFT Review filters focused on the following evidence types: human (epidemiology), animal models for human health, and in vitro studies. The use of SWIFT Review is consistent with the IRIS Handbook [4] and the PFAS systematic review protocol [5]. For all literature searches, the evidence stream filters used were human, animal (all), animal (human health model), [no tag], epidemiological quantitative analysis, and in vitro (with one exception – for the 2022 and 2023 literature searches, the in vitro evidence stream filter was not used because the goal of those literature search was to identify studies relevant to dose-response only). Studies not captured using these filters were not considered further. Studies that were captured with these SWIFT Review evidence stream filters were exported as a Research Information System file for title and abstract screening using either DistillerSR or SWIFT ActiveScreener software (described in subsequent sections of this appendix).

Following SWIFT Review filtering, literature search results were imported into either DistillerSR (Evidence Partners; https://www.evidencepartners.com/products/distillersr-systematic-review-software) or SWIFT ActiveScreener (Sciome; https://www.sciome.com/swift-activescreener/) software and were screened against the PECO criteria at the title and abstract level to identify PECO-relevant studies. Studies that met the PECO criteria were tagged as having relevant human data, relevant animal data (in a mammalian model), or a physiologically based pharmacokinetic model. Studies that did not meet the PECO criteria as determined by title/abstract screening but did appear to include potentially important supplemental information were categorized according to the type of supplemental information they provided.

# Supplemental Table 3. Populations, Exposure, Comparator, and Outcome (PECO) Criteria and Study Eligibility Criteria.

| PECO element | Evidence |
| --- | --- |
| Populations | Mother-child pairs who participated in a cohort, case-control or cross-sectional study used to examine birth weight in relation to exposure to perfluoroctane sulfonic acid (PFOS) with a blood measure for exposure obtained either before, at the time of, or up to 30 days after birth. |
| Exposures | **Measured concentration of PFOS in serum or plasma, whole blood, or dried neonatal blood spot.** |
| Comparators | Difference in birth weight (grams) per ng/mL serum PFOS or per log ng/mL serum PFOS or a result that can be re-expressed in those terms. |
| Outcomes | Birth weight measurements expressed in grams or a unitless standardized measure such as a Z-score. |

## Study Inclusion and Exclusion

Following full text screening, we identified 72 publications examining birth weight measures in relation to PFOS exposure biomarkers. Studies were included in our meta-analysis if they: 1) reported regression coefficients (i.e., “betas” or βs) for the association between BW changes and PFOS in both sexes individually or combined; 2) reported 95% confidence intervals (CIs) or other measures of variance such as a standard error or a p-value that allowed for CI estimation; 3) measured concentrations in maternal blood before, during or after pregnancy, or infant umbilical cord or heel stick collected after pregnancy. There were 53 distinct units of observation in the primary meta-analysis from 54 publications and 50 observations were used in the meta-regression.

To avoid overweighting results from different analyses of the same cohort across multiple publications, the analysis with the largest sample size was used as the unit of observation. For example, the first study listed here in each bullet were selected among the multiple publications from the same cohort were identified:

-Aarhus Birth Cohort: Bach et al. [52]; Bjerregaard-Olesen et al. [78]

-Atlanta African American Maternal-Child Cohort: Eick et al. [57]; Taibl et al. [79]

-Guangzhou Birth Cohort Study: Chu et al. [55]; Li et al. [80]

-Hangzhou, China cohort: Shen et al. [64]; Tian et al. [81]

-Health Outcomes and Measures of the Environment Cohort: Shoaff et al. [65, 82]; Woods et al. [83]

-Hokkaido Birth Cohort Study: Kashino et al. [39]; Kishi et al. [84]; Kobayashi et al. [85]; Minatoya et al. [86]

-POPUP COHORT: Swedish Environmental Protection Agency [87]; Gyllenhammar et al. [36]

-SELMA: Wikström et al. [70]; Gennings et al. [88]

-Shanghai Birth Cohort: Wang et al. [68]; Chen et al. [34]

-UPSTATE KIDS cohort: Bell et al. [53]; Yeung et al. [89]

Another recent publication by Padula et al. [75] overlaps with several included studies from The Environmental Influences on Child Health Outcomes (ECHO) wide cohort [33, 57, 63, 66]. Given the disparate sampling times across ECHO Cohorts, Padula et al. [33] was not included in our primary analysis but was considered in a sensitivity analysis.

We obtained additional mean BW results from other related publications (e.g., Swedish Environmental Protection Agency [87] and from study authors [82]. One publication [90] was excluded because the study did not report the requisite data; correspondence with study authors requesting additional data went unanswered. Non-replies also precluded three studies from being in our meta-regression although there was enough data for their inclusion in the meta-analysis [30, 46, 64].

Following exclusion of these 14 studies (13 duplicates and 1 study with missing data), 58 publications examining 57 study populations underwent study quality evaluation. The analysis of boys in Marks et al. [91] and girls in Maisonet et al. [42] were from the same cohort (Avon Longitudinal Study of Parents and Children) study. Thus, Maisonet et al. [42] is used here as a label to include the pooled sex-specific results based on both of these publications. Three other sex-specific studies [45, 51, 60] were pooled individually using inverse-variance weighting which provided an effect estimate for the overall study population. For comparability with other publications that combined country-specific estimates (e.g., [41]) data from Norway and Sweden in Lauritzen et al. [59] were also pooled together. For the primary analysis, we excluded three studies [92-94] that met the inclusion criteria but were considered *uninformative* due to critical study deficiencies in some risk of bias domains (e.g., confounding; analysis) or multiple domain deficiencies. Thus, there were 53 distinct units of observation in the primary meta-analysis from 54 publications and 50 observations were used in the meta-regression.

For stratified and sensitivity analyses, the following subgroups are used and results described in Table 2:

14 studies with re-expression of β: Bach et al. [52], Bell et al. [53], Buck Louis et al. [54], Cao et al. [23], de Cock et al. [35], Eick et al. [57], Gao et al. [25], Gardener et al. [10], Hjermitslev et al. [38], Maisonet et al. [42], Sagiv et al. [63], Sevelsted et al. [46], Shoaff et al. [65], Whitworth et al. [69].

12 studies without log based β: Bach et al. [52], Cao et al. [23], de Cock et al. [35], Eick et al. [57], Gao et al. [25], Gardener et al. [10], Hjermitslev et al. [38], Maisonet et al. [42], Sagiv et al. [63], Sevelsted et al. [46], Shoaff et al. [65], Whitworth et al. [69].

10 cord serum or plasma studies: Apelberg et al. [31], Cao et al. [23], Chen et al. [34], de Cock et al. [35], Govarts et al. [58], Kwon et al. [40], Shi et al. [27], Wang et al. [48], Wang et al. [68], Xu et al. [29].

16 Asian studies: Cai et al. [32]. Cao et al. [23], Chen et al. [34], Chu et al. [55], Gao et al. [25], Kashino et al. [39], Kwon et al. [40], Luo et al. [61], Shen et al. [64], Shi et al. [27], Wang et al. [48], Wang et al. [49], Wang et al. [68], Xu et al. [29], Yao et al. [11], Zhang et al. [30].

9 earliest of the early subgroup studies with gestational age <=10 weeks: Ashley-Martin et al. [51], Meng et al. [43], Sagiv et al. [63], Siwakoti et al. [47], Wikström et al. [70], Darrow et al. [56], Lind et al. [60], Robledo et al. [45], Zhang et al. [72].

13 earliest of the early subgroup studies with gestational age <=13 weeks and excluding studies sampled at preconception: Ashley-Martin et al. [51], Bach et al. [52], Buck Louis et al. [54], Chang et al. [33], Darrow et al. [56], Lind et al. [60], Manzano-Salgado et al. [62], Meng et al. [43], Robledo et al. [45], Sagiv et al. [63], Siwakoti et al. [47], Wikström et al. [70], Zhang et al. [72].

4 studies that were replaced by Padula et al. [75]: Chang et al. [33]; Eick et al. [57]; Sagiv et al. [63]; Starling et al. [66]; β of Padula et al. [75] was -12.8 g (95%CI: -30.6, 5.1) per IQR ng/mL.

8 studies with z-score BW data converted to mean differences: Cai et al. [32], Espindola Santos et al. [24], Gardener et al. [10], Sevelsted et al. [46], Siwakoti et al. [47], Wang et al. [49], Wang et al. [68], Xiao et al. [71].

6 studies that reported quantile results: Cao et al. [23], de Cock et al. [35], Eick et al. [57], Gao et al. [25], Gardener et al. [10], Maisonet et al. [42].

# Supplemental Table 4. Study Characteristics of 53 informative PFOS studies included from 54 publications.

| **Reference** | **Study name and years** | **Country** | **Study design** | **n** | **Study confidence** | **Matrix** | **Exposure window** | **Adjustment factors** |
| --- | --- | --- | --- | --- | --- | --- | --- | --- |
| Apelberg et al., 2007 | Baltimore THREE Study (2004-2005) | United States | Cross-sectional | 293 | Medium | Cord blood | Birth | gestational age, maternal age, BMI, race, parity, smoking, baby sex, height, net weight gain, diabetes, hypertension |
| Ashley-Martin et al., 2017 | Maternal-Infant Research on Environmental Chemicals (MIREC) (2008-2011) | Canada | Cohort | 1509 | High | Maternal blood | T1 | maternal age, prepregnancy BMI, parity, household income, smoking |
| Bach et al., 2016 | Aarhus Birth Cohort (2008-2013) | Denmark | Cohort | 1507 | High | Maternal serum | T1-2 | education, maternal age, maternal prepregnancy BMI |
| Bell et al., 2018 | Upstate KIDS (2008-2010) | United States | Cross-sectional | 2071 | High | Blood (dried) | Birth | maternal age, maternal BMI, maternal education, infertility treatment, parity |
| Buck Louis | NICHD Fetal Growth Studies (2009-2013) | United States | Cohort | 2106 | High | Maternal plasma | T1 | age, education, pre-pregnancy BMI, infant sex, serum cotinine |
| Cai et al., 202 3 | Maoming Birth Cohort (2015-2018) | China | Cohort | 202 | Medium | Maternal serum | T2-3 | maternal age, pre-BMI, environmental tobacco exposure during pregnancy, infant gender, gestational age, family income |
| Callan et al., 2016 | AMETS (2008-2011) | Australia | Cross-sectional | 98 | Low | Maternal blood | T3 | gestational age, maternal height, prepregnancy BMI, weight gain during pregnancy, sex of infant |
| Cao et al., 2018 | Zhoukou (2013-2015) | China | Cohort | 337 | Low | Cord blood serum | Birth | maternal age, maternal education, household income, parity, smoking of father, drinking of father, infant's gender |
| Chang et al., 2022 | Emory University African American Vaginal, Oral, and Gut Microbiome in Pregnancy Study (2014-2018) | United States | Cohort | 370 | Medium | Maternal serum | T1-2 | maternal age, education, BMI, parity, tobacco use, marijuana use, infant sex |
| Chen et al., 2012 | Taiwan Birth Panel Study (TBPS) (2004-2005) | Taiwan | Cross-sectional | 429 | Medium | Cord blood plasma | Birth | maternal age, prepregnancy BMI, education level, log (Ln)-transformed cord blood cotinine levels, type of delivery, parity and infant sex and gestational age |
| Chen et al., 2021 | Shanghai Birth Cohort (SBC) (2013-2015) | China | Cohort | 214 | Medium | Maternal plasma | T1-2 | maternal age, BMI, educational level, occupation, income, fetal sex, parity, gestational age, smoking, alcohol |
| Chu et al., 2020 | Guangzhou Birth Cohort (GBC) (2013) | China | Cohort | 372 | High | Maternal serum | T3 | gestational age, maternal age, maternal occupation, maternal education, family income, parity, infant sex |
| Darrow et al., 2013 | C8 Health Project and Community Follow-up Study (2005-2011) | United States | Cohort | 1470 | High | Maternal serum | Preconception | maternal age, educational level, smoking status, parity, BMI, self-reported diabetes, time between conception and serum measurement (year strata), indicator variables for gestational week |
| de Cock et al., 2016 | OBELIX (2011-2013) | Netherlands | Cross-sectional | 64 | Medium | Cord blood | Birth | gestational age, maternal BMI, maternal height, maternal age at birth, parity, paternal BMI, paternal height, education, fish intake |
| Eick at al., 2020 | Chemicals in our Bodies (CIOB) cohort (2014-2018) | United States | Cohort | 506 | High | Maternal serum | T1-3 | maternal age, maternal race/ethnicity, pre-pregnancy BMI, maternal education, smoking status, parity, food insecurity |
| Espindola Santos et al., 2021 | PIPA project (2017) | Brazil | Cross-sectional | 72 | Low | Maternal serum | T3 | education, income, race, pre-gestational BMI, smoking active and passive, alcohol consumption, gestational age, primiparity, age (continuous), fish consumption |
| Gao et al., 2019 | Affiliated Hospital of Capital Medical University (2015-2016) | China | Cross-sectional | 132 | Low | Maternal serum, Cord blood serum | T3 | maternal weight, height, BMI, gestational age |
| Gardener et al., 2021 | Vanguard Pilot Study of the National Children’s Study (2009) | United States | Cohort | 403 | High | Maternal serum | T2-3 | maternal age, education, race/ethnicity, pre-pregnancy BMI, prenatal smoking, parity, gestational age at serum collection |
| Govarts et al., 2016 | FLEHS II (2008-2009) | Belgium | Cohort | 213 | High | Cord blood plasma | Birth | gestational age, child sex, smoking of the mother during pregnancy, parity, maternal prepregnancy BMI |
| Gyllenhammar et al., 2018 | Persistent Organic Pollutants in Uppsala Primiparas (POPUP) (1997-2011) | Sweden | Cohort and cross-sectional | 599 | Medium | Maternal serum | Post partum | sampling year, maternal age, pre pregnancy BMI, maternal weight gain during pregnancy, maternal weight loss after delivery, years of education, smoking during pregnancy, total fish consumption |
| Hamm et al., 2010 | Edmonton (2005-2006) | Canada | Cohort | 252 | Medium | Maternal serum | T2 | maternal age, maternal race, gravida, maternal weight and height, smoking, infant gender, gestational age at birth |
| Hjermitslev et al., 2020 | ACCEPT (2010-2011, 2013-2015) | Greenland | Cohort | 256 | Medium | Maternal serum | T1-2 | maternal age, plasma cotinine, parity, alcohol consumption |
| Kashino et al., 2020 | Hokkaido Study on Environment and Children's Health (2003-2009) | Japan | Cohort | 1951 | Medium | Maternal serum | T3 | gestational age, maternal age, pre-pregnancy BMI, parity, infant sex, maternal educational level, plasma cotinine concentration during pregnancy |
| Kwon et al., 2016 | Ewha Birth & Growth Retrospective Cohort (EBGRC) (2006-2010) | Korea | Cross-sectional | 268 | Medium | Cord blood serum | Birth | maternal age, pre-pregnancy BMI, past history of alcohol consumption and child’s gestational age, gender, parity |
| Lauritzen et al., 2017 | National Institute of Child Health and Human Development (NICHD) Scandinavian Successive Small-for-Gestational Age (SGA) (1986-1988) | Sweden, Norway | Cohort | 424 | High | Maternal serum | T2 | maternal age, height, prepregnancy BMI, education, parity, smoking status at conception, interpregnancy interval, offspring sex |
| Lenters et al., 2016 | INUENDO (2002-2004) | Greenland, Ukraine, Poland | Cohort | 1250 | Medium | Maternal serum | T1-3 | study population, maternal age, prepregnancy BMI, parity, gestational age, infant sex, maternal height, alcohol, cotinine, vitamin D |
| Lind et al., 2017 | Odense Child Cohort (OCC) (2010-2012) | Denmark | Cohort | 338 | High | Maternal serum | T1 | gestational age, smoking, parity, BMI |
| Luo et al., 2021 | Guangzhou (2017-2019) | China | Cohort | 224 | High | Maternal plasma | T3 | maternal age, prepregnancy BMI, education, parity, environmental tobacco smoke exposure, alcohol drinking, gestational age, newborn sex |
| Maisonet et al., 2012 | Avon Longitudinal Study of Parents and Children (ALSPAC) (1991-1992) | Great Britain | Cohort | 422 | Medium | Maternal serum | T1-3 | maternal smoking during pregnancy, maternal prepregnancy BMI, previous live births, gestational age |
| Manzano-Salgado et al., 2017 | INfancia y Medio Ambiente Project (INMA) (2003-2008) | Spain | Cohort | 1202 | High | Maternal blood | T1-2 | maternal age, parity, pre-pregnancy BMI, fish intake during pregnancy |
| Meng et al., 2018 | Danish National Birth Cohort (DNBC) (1996-2002) | Denmark | Cohort | 3507 | Medium | Maternal serum | T1-2 | infant sex, infant birth year, gestational week of blood draw, maternal age, parity, socio-occupational status, pre-pregnancy BMI, smoking during pregnancy, alcohol intake during pregnancy, study sample |
| Mwapasa et al, 2023 | Ndirandea_Chiradzulu_Thyolo (2020-2021) | Malawi | Cohort | 508 | Low | Maternal serum | T3/Birth | maternal age, area of residence (urban vs. rural), maternal educational level, parity, source of drinking water |
| Peterson et al., 2022 | MADRES (2015-2019) | United States | Cohort | 342 | Medium | Maternal serum | T1-3 | child’s sex, country of birth, fish consumption, gestational age at birth, gestational age at the time of blood sampling, household income, maternal education, parity, race/ethnicity, pre-pregnancy BMI, maternal age at study recruitment, recruitment site |
| Robledo et al., 2015 | Longitudinal Investigation of Fertility and the Environment (LIFE) (2005-2009) | United States | Cohort | 117 | Medium | Maternal serum | Preconception | maternal and paternal serum lipids, serum cotinine, BMI, maternal age, difference in paternal age, infant gender, individual and partner sum of remaining chemical concentrations in each chemical's respective class |
| Sagiv et al., 2018 | Project Viva (1999-2002) | United States | Cohort | 1644 | High | Maternal blood | T1-2 | maternal age at enrollment, race/ethnicity, education, prenatal smoking, parity, history of breastfeeding, prepregnancy BMI, paternal education, household income, child sex, gestational age at blood draw |
| Sevelsted et al., 2022 | COPSAC-2010 (2008–2010) | Denmark | Cohort | 653 | Medium | Maternal plasma | T2-Postpartum | parity, race, maternal social circumstances, maternal prepregnancy BMI, maternal height, maternal biomarker for fish intake, pregnancy fish oil supplementation RCT, birth address urbanicity |
| Shen et al., 2022 | Hangzhou Women’s Hospital of the School of Medicine (2020-2021) | China | Cohort | 506 | High | Maternal serum | T3 | maternal age, prenatal BMI, education, occupation, smoking, alcohol drinking, ethnicity, delivery mode, parity |
| Shi et al., 2017 | Haidon Hospital (2012) | China | Cross-sectional | 170 | Low | Cord blood serum | Birth | maternal age, pre-pregnancy BMI, parity, gestation age, fetus gender |
| Shoaff et al., 2018 | Health Outcomes and Measures of the Environment (HOME) Study (2003-2006) | United States | Cohort | 345 | High | Maternal blood | T2-3/Birth | maternal age at delivery, race, marital status, insurance, income, education, parity, serum cotinine, depressive symptoms, mid-pregnancy BMI, food security, fruit/vegetable and fish consumption during pregnancy, prenatal vitamin use |
| Siwakoti et al., 2023 | LIFECODES (2006-2008) | United States | Case-control | 477 | Medium | Maternal plasma | T1-2 | maternal age, race, insurance status, educational status, prepregnancy BMI, parity |
| Starling et al., 2017 | Healthy Start (2009-2014) | United States | Cohort | 628 | High | Maternal serum | T2-3 | maternal age, pre-pregnancy BMI, race/ethnicity, education, gestational weight gain, smoking during pregnancy, gravidity, gestational age at blood draw, infant sex, gestational age at birth |
| Valvi et al., 2017 | Faroe Islands (1997-2000) | Faroe Islands | Cohort | 604 | High | Maternal serum | T3 | maternal age at delivery, education, parity, pre-pregnancy BMI, smoking during pregnancy, child sex |
| Wang et al., 2019 | Maternal and Child Health Care Hospital of Tangshan City (2013) | China | Cohort | 223 | Medium | Cord serum | Birth | pregnant age, family income, maternal education level, maternal career, husband's smoking, energy daily intake, daily physical activity, gestational age, parity, pre-pregnant maternal BMI, gestational diabetes mellitus, infant sex, delivery mode, gestational weight gain |
| Wang et al., 2023a | Shanghai Birth Cohort (2013-2016) | China | Cohort | 1405 | High | Maternal plasma | T1-2 | maternal age, maternal educational level, pre-pregnancy BMI, smoking status, fish or seafood intake, estimated glomerular filtration rate, parity |
| Wang et al., 2023b | Sheyang Mini Birth Cohort Study (2009-2010) | China | Cross-sectional | 1087 | Medium | Cord serum | Birth | maternal age, maternal education level, maternal weight gain during pregnancy, prepregnancy BMI, family annual income, gestational age, passive smoke during pregnancy, infant sex, parity |
| Whitworth et al., 2012 | Norwegian Mother and Child Cohort Study (MoBa) (2003-2004) | Norway | Cohort | 838 | High | Maternal plasma | T2 | maternal age, prepregnancy BMI, parity, albumin concentration, maternal education, interpregnancy interval, quadratic interpregnancy interval, consumption of lean fish |
| Wikström et al., 2020 | Swedish Environmental Longitudinal, Mother and child, Asthma and allergy (SELMA) (2007-2010) | Sweden | Cohort | 1533 | High | Maternal serum | T1-2 | gestational age, sex, maternal weight, parity, cotinine levels |
| Workman et al., 2019 | Canadian Healthy Infant Longitudinal Development Study (2010-2011) | Canada | Cohort | 414 | Low | Maternal plasma | T2-3 | maternal age, smoking during pregnancy, high blood pressure during pregnancy, diabetes during pregnancy, parity, infant sex, gestational age |
| Xiao et al., 2020 | Faroe Islands (1994-1995) | Denmark | Cohort | 171 | High | Maternal blood | T3 | child sex, parity, maternal BMI, maternal height, maternal education, maternal age, smoking and drinking alcohol during pregnancy, total PCB, mercury |
| Xu et al., 2019 | Women’s Hospital of the School of Medicine (2016-2017) | China | Cross-sectional | 98 | Low | Cord blood serum | Birth | maternal age, maternal BMI, pregnancy weight gain, gestation age, education, job, abortion times, parity times, birth gender, drinking water |
| Yao et al., 2021 | Laizhou Wan Birth Cohort (LWBC) (2010-2013) | China | Cross-sectional | 369 | High | Maternal serum | T3 | maternal age, maternal education, pre-pregnancy maternal BMI, parity |
| Zhang et al., 2022 | Qingyuan City (2016) | China | Cohort | 94 | Low | Maternal serum | T3 | maternal age, gestational age |
| Zhang et al., 2024 | Environment and Reproductive Health (EARTH) Study (2005-2019) | United States | Cohort | 312 | High | Maternal serum | Preconception | maternal age, BMI, education, race, smoking history, type of medically assisted reproduction, study period by five years intervals |
| Zheng et al., 2024 | US Collaborative Perinatal Project (1960-1966) | United States | Cohort | 97 | Medium | Maternal serum | T2-3 | maternal age at pregnancy, weight change in pounds during pregnancy, duration of gestation in days, number of cigarettes smoked per day during pregnancy, marital status, parity, race, family socioeconomic index, child sex, year of blood collection |

Abbreviations: n: sample size; BMI: body mass index T: Trimester (e.g. T1: Trimester 1); PCBs: polychlorinated biphenyls.

# Supplemental Table 5. Central Tendency Sample Timing Estimation Procedures and Notes.

| **Study** | **Sampling Window** | **Measure of Central Tendency (weeks)^1^** | **Other Reported Statistics (weeks)** | **Detail on Data Source, Reporting, and Calculations** |
| --- | --- | --- | --- | --- |
| ***Early Pregnancy*^2^** | |  |  |  |
| **Ashley-Martin et al., 2017** | Trimester 1 | 9.9 (calculated) | None | To estimate a measure of central tendency, the first trimester midpoint was calculated, assuming no sampling before six weeks. This gives a range of 6 weeks to 13 weeks and 6 days, and a midpoint of 9.9 weeks. |
| **Bach et al., 2016** | Trimester 1 & 2 | 12 (mode) | 9-20 (range) | The study authors reported that most samples were collected at 12 weeks; and that 96% of samples were collected within 13 weeks. |
| **Buck Louis et al., 2018** | Trimester 1 | 11.9 (midpoint) | 10-13.9 (range) | To estimate a measure of central tendency, the midpoint of the range (10 to 13.9 weeks) was calculated: 11.9 weeks. |
| **Chang et al., 2022** | Trimester 1 & 2 | 11.4 (median) | 11.3 (mean)  9.6-12.6 (IQR)  8.1-14.6 (range) | The study authors reported the median (11.4 weeks) and range (9.6 to 14.6 weeks) via email (D. Liang, personal communication, November 29, 2022). |
| **Darrow et al., 2013** | Preconception | 0 (assumed) | None | Because samples were collected preconception, the measure of central tendency is assumed to be 0 weeks. |
| **Eick et al., 2020** | Trimester 1, 2 & 3 | 20 (median) | 12-28 (range) | Median value was reported by study authors via email (S. Eick, personal communication, April 26, 2022). |
| **Hjermitslev et al., 2020** | Trimester 1, 2 & 3 | 15.39 (calculated) | 7-40 (range) | This study was assigned to the *early* strata because sampling predominantly occurred earlier in pregnancy: study authors report that the mean gestational week of sampling in 2010–2011 was week 26.2, and in 2013–2015 all samples were collected before the end of week 13. 38% of samples were taken in 2010-2011; 62% were collected in 2013-2015 (Bonefeld-Jørgensen et al., 2022).  To estimate a measure of central tendency, the weighted mean of midpoints was calculated: 15.39 weeks. |
| **Lind et al., 2017** | Trimester 1 | 10 (median) | 5-12 (range) |  |
| **Maisonet et al., 2012** | Trimester 1, 2 & 3 | 15 (median) | 10-28 (IQR) | The analysis of boys in Marks et al. (2019) and girls in Maisonet et al. (2012) were from the same cohort, ALSPAC. Thus, Maisonet et al. (2012), which used earlier pregnancy sampling, is used to denote the combined sex-specific results. |
| **Manzano-Salgado et al., 2017** | Trimester 1, 2 & 3 | 12.3 (mean) | 5.6 (SD) | Sampling was reported to be in the first trimester (Manzano-Salgado et al., 2017), but supporting information showed some sampling outside of the first trimester occurred (per author, see Manzano-Salgado et al, 2016-doi: 10.1016/j.envint.2016.04.004). However, first trimester sampling was predominant, so this study is designated as “early”. |
| **Meng et al., 2018** | Trimester 1 & 2 | 8 (mean) | 4-14 (range) | The mean is reported in related publication (Liew et al., 2020). |
| **Peterson et al., 2022a** | Trimester 1, 2 & 3 | 19.1 (median) | 5.7-38.3 (range) |  |
| **Robeldo et al., 2015** | Preconception | 0 (assumed) | None | Because samples were collected preconception, the measure of central tendency is assumed to be 0 weeks. |
| **Sagiv et al., 2018** | Trimester 1 & 2 | 9 (median) | 5-19 (range) |  |
| **Siwakoti et al., 2023** | Trimester 1 & 2 | 9.86 (median) | 5.14-21.6 (range) |  |
| **Wang et al., 2023a** | Trimester 1 & 2 | 15 (mean) | 9-16 (range) |  |
| **Wikström et al., 2020** | Trimester 1 & 2 | 10 (median) | None |  |
| **Zhang et al., 2024** | Preconception | 0 (assumed) | None | Because samples were collected preconception, the measure of central tendency is assumed to be 0 weeks. |
| ***Late-pregnancy*^3^** | |  |  |  |
| **Cai et al., 2023** | Trimester 3 | 38.95 (calculated) | None | Study authors clarified via email that all samples were collected from mothers prior to delivery, and all samples collected in trimester 3 (X. Zeng, personal communication, July 1, 2014). To estimate a measure of centrality, we used the median delivery date of 39.1 weeks and the median sample collection time of 1.0 day before delivery. |
| **Callan et al., 2016** | Trimester 3 | 37.7 (calculated) | None | Samples were taken two weeks before due dates, so to calculate the central tendency and spread, two weeks was subtracted from the average due date and range, 39.7 weeks (35 to 42 weeks): 37.7 weeks (33 to 40 weeks). |
| **Chu et al., 2020** | Trimester 3 | 33.3 (calculated) | None | To estimate a measure of centrality, the midpoint of the third trimester was calculated assuming the third trimester begins at 28 weeks and ends at the average reported gestational age at birth, 38.6 weeks (1.7 SD): 33.3 weeks. |
| **Espindola Santos et al., 2021** | Trimester 3 | 30 (midpoint) | 28-32 (range) | To estimate a measure of central tendency, the midpoint of the range (28 to 32 weeks) was calculated: 30 weeks. |
| **Gao et al., 2019** | Trimester 3 | 39 (median) | 38.8 (mean)  1.32 (SD)  31-41 (range) |  |
| **Gardener et al., 2021** | Trimester 2 & 3 | 32 (median) | 31.9 (mean)  3.3 (SD)  29-34 (IQR)  20-40 (range) | All statistics were reported by study authors via email (H. Gardener, personal communication, May 19, 2022). |
| **Hamm et al., 2010** | Trimester 2 | 15.5 (midpoint) | 15-16 (range) | To estimate a measure of central tendency, the midpoint of the range (15 to 16 weeks) was calculated: 15.5 weeks. |
| **Kashino et al., 2020** | Trimester 3 | 29 (median) | None |  |
| **Lauritzen et al., 2017** | Trimester 2 | 18.5 (midpoint) | 17-20 (range) | To estimate a measure of central tendency, the midpoint of the range (17 to 20 weeks) was calculated: 18.5 weeks. |
| **Lenters et al., 2016** | Trimester 1, 2 & 3 | 25.2 (calculated) | 33, 25, 23 (medians)  4-40 (range) | Study authors reported country-specific medians: 33 weeks (Poland, 18%), 25 weeks (Greenland, 32%), 23 weeks (Ukraine, 49%). To estimate a measure of centrality, a weighted mean of country-specific medians was calculated: 25.2 weeks. Given that late pregnancy sample sampling was predominant, this is designated as a “late” sample timing study. |
| **Luo et al., 2021** | Trimester 3 | 39.3 (mean) | None | The study authors note that all samples were collected within three days of giving birth. |
| **Mwapasa et al., 2023** | Trimester 3/At Birth | 37.38 (calculated) | None | The study authors report that sampling occurred during the third trimester. They also clarify that most samples were taken 24-48 hours before delivery, although some may have been up to 12 hours after birth; so, this is classified as late pregnancy. The authors report that the average gestational age at birth was 37.59 weeks (1.53 SD). To estimate a measure of central tendency, we subtracted 24 hours and 48 hours from the average birth age (37.3 and 37.45, respectively) and calculated the average: 37.38 weeks. |
| **Sevelsted, et al., 2022** | T2-Postpartum | Not Available | None | Two different PFOS samples were averaged from week 24 and 1 week postpartum. |
| **Shen et al., 2022** | Trimester 3 | Not Available | None | Samples were collected a few days before delivery. |
| **Shoaff et al., 2018** | Trimester 2 & 3,  At Birth | 18.1 (calculated) | 16, 26, 40 (exact) | The authors reported that 86%, 9%, and 5% of samples were taken at 16, 26, and 40 weeks, respectively. Also, this study was assigned to the *late* strata instead of *post* because only 5% of samples taken at delivery and a sensitivity analysis showed results to be robust to second trimester sampling. To estimate a measure of central tendency, a weighted mean was calculated: 18.1 weeks. |
| **Starling et al., 2017** | Trimester 2 & 3 | 27 (median) | 20-34 (range) |  |
| **Valvi et al., 2017** | Trimester 3 | 34 (exact)^4^ | None |  |
| **Whitworth et al., 2012** | Trimester 2 | 18 (mean) | 12-37 (range) |  |
| **Workman et al., 2019** | Trimester 2 & 3 | 28.6 (median) | 27.7 (mean)  14.3-39.6 (range) | All statistics are provided by the study authors via email (C. Workman, personal communication, 11/21/2022). |
| **Xiao et al., 2020** | Trimester 3 | 34 (exact)^4^ | None |  |
| **Yao et al., 2021** | Trimester 3 | 39.4 (mean) | None |  |
| **Zhang et al., 2022** | Trimester 3 | Not Available | None |  |
| **Zheng et al., 2024** | Trimester 2 & 3 | 32.3 (median) | 26.3-41.4 (range) | All statistics are provided by the study authors via email (S. Buka, personal communication, 04/05/2024). |
| ***Post-pregnancy*^5^** | |  |  |  |
| **Apelberg et al., 2007** | At Birth | 39.29 (median) | 38.86 (mean) |  |
| **Bell et al., 2018** | After Birth | 39.0 (median) | 38.78 (mean)  26-42 (range) | Samples were collected between 24 hours postpartum and the time of hospital discharge. All statistics are provided by the study authors via email (E. Bell, personal communication, March 7, 2022). |
| **Cao et al., 2018** | At Birth | 40 (assumed) | None | Samples collected at birth, but no statistics were reported; the measure of central tendency is assumed to be 40 weeks since this is the typical assumed length of gestation in the U.S. (NIH, 2017). |
| **Chen et al., 2012** | At Birth | 39.8 (median) | 29-41 (range) |  |
| **de Cock et al., 2016** | At Birth | 39.8 (mean) | 1.3 (SD) |  |
| **Govarts et al., 2016** | At Birth | 40 (median) | 34-42 (range) | The study authors report that all samples were taken postpartum. Since the minimum sample time is 34 weeks, this indicates that some infants included in this study may have been born pre-term (i.e., less than 37 weeks). |
| **Gyllenhammar et al., 2018** | After Birth | 43 (calculated) | None | Samples were taken three weeks after delivery, which occurred on average at 40 weeks (range: 34.9 to 43.1 weeks). To calculate the average sampling time and range, three weeks was added to the mean and range of delivery times: 43 weeks (37.9 to 46.1 weeks). |
| **Kwon et al., 2016** | At Birth | 40 (assumed) | None | Because samples were collected at birth, but no statistics were reported; the measure of central tendency is assumed to be 40 weeks. |
| **Shi et al., 2017** | At Birth | 39.48 (mean) | 4.23 (SD) |  |
| **Wang et al., 2019** | At Birth | 40 (assumed) | None | Because samples were collected at birth, but no statistics were reported; the measure of central tendency is assumed to be 40 weeks. |
| **Wang et al., 2023b** | At Birth | 39.3 (mean) | 1.2 (SD) |  |
| **Xu et al., 2019** | At Birth | 39.39 (mean) | 1.37 (SD) |  |

Abbreviations: IQR = interquartile range; SD = standard deviation.

^1^If multiple measures of central tendency are reported, the median was preferred over the mean.

^2^The early group included studies with biomarker samples collected pre-conception or from earlier stages of pregnancy, i.e., measurements were taken exclusively in trimester 1, or a combination of trimesters 1 and 2, or trimesters 1-3.

^3^The late group included studies with biomarker sampling from the middle and/or later stages of pregnancy, i.e., exclusively from or a combination of samples from trimesters 2 or 3, or a combination these trimesters along with any measures at or after delivery.

^4^Exact means all samples collected at gestational weeks 34.

^5^The post-pregnancy group included studies with biomarker samples collected exclusively from post-birth or at delivery.

# Supplemental Table 6: Results from meta-regression analyses for 50 studies

| **Grouping** | **Potential moderator^a^** | **Change in birth weight (95%CI)** | **Total heterogeneity (I^2^)** | | **Overall Heterogeneity explained (R^2^)** | | **Residual heterogeneity test (Qep)** | | **Permutation test of moderators (QMp)** | |  |
| --- | --- | --- | --- | --- | --- | --- | --- | --- | --- | --- | --- |
| All studies |  |  |  |  | |  | |  | |  |  |
|  | meta-effect^b^ | -30.9 (-42.5, -19.3) | 43.41 |  | |  | |  | |  |  |
|  | per week increase in central tendency | 0.10 (-0.09, 0.29) | 42.85 | 0.00 | | >0.001 | | 0.34 | |  |  |
|  | Asia | 3.1 (-26.7, 32.8) | 40.08 | 11.44 | | >0.001 | | 0.26 | |  |  |
|  | North America | 21.6 (-7.1, 50.2) |  |  | |  | |  | |  |  |
|  | Not adjusted for maternal age | 18.0 (-32.1, 68.0) | 46.55 | 0.00 | | >0.001 | | 0.52 | |  |  |
|  | Not adjusted for gestational age | 15.0 (-7.9, 37.9) | 39.74 | 8.57 | | >0.001 | | 0.20 | |  |  |
|  | Not adjusted for other PFAS | -14.0 (-38.9, 11.0) | 39.51 | 5.89 | | >0.001 | | 0.27 | |  |  |
|  | Not adjusted for pregnancy weight gain | -9.4 (-40.78, 22.0) | 43.09 | 0.00 | | >0.001 | | 0.58 | |  |  |
|  | Median PFOS in study | -1.4 (-2.9, 0.1) | 43.92 | 8.23 | | >0.001 | | 0.09 | |  |  |
|  | Mean birthweight in study | -0.8 (-7.3, 5.8) | 42.30 | 0.00 | | >0.001 | | 0.84 | |  |  |
|  | Not adjusted for parity | 14.7 (-28.3, 57.7) | 44.92 | 0.00 | | >0.001 | | 0.49 | |  |  |
|  | Not adjusted for BMI | -34.3 (-76.9, 8.3) | 41.09 | 8.87 | | >0.001 | | 0.14 | |  |  |
|  | Not adjusted for GFR | -6.3 (-40.5, 27.9) | 45.19 | 0.00 | | >0.001 | | 0.73 | |  |  |
| All studies adjusted for central tendency of sample timing |  |  |  |  | |  | |  | | | |
|  | Asia | -4.8 (-35.3, 25.7) | 35.61 | 24.71 | | >0.001 | | 0.26 | |  |  |
|  | North America | 22.1 (-5.6, 49.8) |  |  | |  | |  | |  |  |
|  | Not adjusted for maternal age | 19.9 (-30.4, 70.2) | 46.07 | 0.00 | | >0.001 | | 0.52 | |  |  |
|  | Not adjusted for gestational age | 19.4 (-3.5, 42.4) | 36.03 | 19.77 | | >0.001 | | 0.20 | |  |  |
|  | Not adjusted for other PFAS | -16.6 (-41.5, 8.3) | 37.23 | 11.92 | | >0.001 | | 0.27 | |  |  |
|  | Not adjusted for pregnancy weight gain | -1.8 (-38.1, 34.5) | 44.27 | 0.00 | | >0.001 | | 0.58 | |  |  |
|  | Median PFOS in study | -1.2 (-2.8, 0.3) | 44.68 | 1.92 | | >0.001 | | 0.09 | |  |  |
|  | Mean birthweight in study | -1.2 (-7.8, 5.4) | 41.54 | 0.00 | | >0.001 | | 0.84 | |  |  |
|  | Not adjusted for parity | 16.0 (-27.1, 59.2) | 44.23 | 0.00 | | >0.001 | | 0.49 | |  |  |
|  | Not adjusted for BMI | -34.3 (-77.0, 8.3) | 40.51 | 7.94 | | >0.001 | | 0.14 | |  |  |
|  | Not adjusted for GFR | -8.3 (-42.8, 26.2) | 44.52 | 0.00 | | >0.001 | | 0.73 | |  |  |
| Log-based studies |  |  |  |  | |  | |  | | | |
|  | meta-effect^b^ | -29.6 (-42.4, -16.8) | 45.11 |  | |  | |  | |  |  |
|  | per week increase in central tendency | 0.1 (-0.1, 0.3) | 44.89 | 0.00 | | >0.001 | | 0.42 | |  |  |
|  | Asia | -8.1 (-42.8, 26.5) | 43.65 | 3.85 | | >0.001 | | 0.33 | |  |  |
|  | North America | 14.4 (-19.7, 48.5) |  |  | |  | |  | |  |  |
|  | Not adjusted for gestational age | 11.9 (-13.9, 37.7) | 42.53 | 2.19 | | >0.001 | | 0.40 | |  |  |
|  | Not adjusted for other PFAS | -16.2 (-42.6, 10.2) | 41.14 | 5.31 | | >0.001 | | 0.25 | |  |  |
|  | Not adjusted for pregnancy weight gain | -8.2 (-40.4, 24.0) | 45.13 | 0.00 | | >0.001 | | 0.62 | |  |  |
|  | Median PFOS in study | -1.0 (-2.8, 0.9) | 48.33 | 0.90 | | >0.001 | | 0.33 | |  |  |
|  | Mean birthweight in study | -0.2 (-7.2, 6.9) | 44.35 | 0.00 | | >0.001 | | 0.96 | |  |  |
| Log-based studies adjusted for central tendency of sample timing |  |  |  |  | |  | |  | | | |
|  | Asia | 15.4 (-17.2, 48.0) | 38.10 | 21.08 | | >0.001 | | 0.24 | |  |  |
|  | North America | 16.6 (-9.7, 42.9) | 39.65 | 10.37 | | >0.001 | | 0.24 | |  |  |
|  | Not adjusted for gestational age | -19.6 (-46.1, 7.0) | 38.69 | 11.53 | | >0.001 | | 0.37 | |  |  |
|  | Not adjusted for other PFAS | -1.6 (-38.7, 35.5) | 46.58 | 0.00 | | >0.001 | | 0.28 | |  |  |
|  | Not adjusted for pregnancy weight gain | -0.8(-2.7, 1.1) | 49.19 | 0.00 | | 0.00003 | | 0.71 | |  |  |
|  | Median PFOS in study | -0.7 (-8.0, 6.5) | 44.36 | 0.00 | | 0.00013 | | 0.53 | |  |  |
|  | Mean birthweight in study | 15.3 (-28.7, 59.3) | 46.62 | 0.00 | | 0.00001 | | 0.70 | |  |  |
| Non-reexpressed studies | meta-effect^b^ | -31.2 (-45.3, -17.1) | 48.82 |  | |  | |  | |  |  |
|  | per week increase in central tendency | 0.1 (-0.1, 0.3) | 48.34 | 0.00 | | 0.00001 | | 0.43 | |  |  |
|  | Asia | -8.9 (-45.7, 27.8) | 47.33 | 0.07 | | 0.00013 | | 0.41 | |  |  |
|  | North America | 14.1 (-23.9, 52.0) | 47.33 | 0.07 | | 0.00013 | | 0.41 | |  |  |
|  | Not adjusted for gestational age | 10.7 (-18.9, 40.2) | 46.12 | 0.00 | | 0.00009 | | 0.46 | |  |  |
|  | Not adjusted for other PFAS | -19.9 (-48.9, 9.2) | 43.24 | 9.38 | | 0.00034 | | 0.20 | |  |  |
|  | Not adjusted for pregnancy weight gain | -8.9 (-43.1, 25.3) | 48.59 | 0.00 | | 0.00001 | | 0.61 | |  |  |
|  | Median PFOS in study | -0.9 (-2.9, 1.0) | 52.38 | 0.47 | | 0.00003 | | 0.38 | |  |  |
|  | Mean birthweight in study | 0.2 (-7.4, 7.8) | 49.15 | 0.00 | | 0.00006 | | 0.96 | |  |  |
| Non-reexpressed studies adjusted for central tendency of sample timing |  |  |  |  | |  | |  | | | |
|  | Asia | 15.5 (-20.8, 51.8) | 41.81 | 16.67 | | 0.00041 | | 0.27 | |  |  |
|  | North America | 15.6 (-14.6, 45.7) | 43.46 | 5.65 | | 0.00018 | | 0.27 | |  |  |
|  | Not adjusted for gestational age | -24.5 (-53.3, 4.4) | 39.07 | 20.53 | | 0.00078 | | 0.46 | |  |  |
|  | Not adjusted for other PFAS | -2.0 (-41.3, 37.3) | 49.97 | 0.00 | | 0.00001 | | 0.21 | |  |  |
|  | Not adjusted for pregnancy weight gain | -0.8 (-2.8, 1.2) | 52.98 | 0.00 | | 0.00002 | | 0.74 | |  |  |
|  | Median PFOS in study | -0.4 (-8.2, 7.5) | 49.21 | 0.00 | | 0.00006 | | 0.58 | |  |  |
|  | Mean birthweight in study | 9.0 (-52.4, 70.4) | 50.64 | 0.00 | | 0.00001 | | 0.73 | |  |  |

Abbreviations: PFAS = per- and polyfluoroalkyl substances; PFOS = perfluorooctane sulfonate; BMI = Body Mass Index; GFR = glomerular filtration rate.

^a^Referents and unit changes for potential moderators are: continent, Europe; adjustment variables, adjusted or considered in study design; mean PFOS is per 1 ln-unit change, centered at 7.5; mean birthweight is per 100 g change, centered at 3405; central tendency of sample timing is per 1 week change in gestational age, centered at 26 weeks. Requirement of 5 studies in each category was not met for adjustment variables of maternal age, BMI, parity, or GFR for subgroups.

^b^meta-effect is the effect estimate from meta-analysis for the grouping, along with the total observed heterogeneity for the grouping

I^2^: Residual heterogeneity across studies after accounting for potential modifiers

R^2^: Amount of heterogeneity explained by potential modifiers

Q_ep_: Test of significance for residual heterogeneity

Q_Mp_: Permutation test to assess statistical significance of potential modifiers

# Additional Supplemental Materials

## Conversion of Categorical Exposure Results to Continuous

We used a method for re-expressing and combining a series of beta coefficients for non-referent exposure categories as documented in previous meta-analyses [3, 15, 16], wherein the re-expressed single beta coefficient had the units ∆ outcome unit/∆ exposure unit and an associated approximate standard error.

Step 1 was used to assign an exposure contrast to each non-referent exposure category as compared with the referent exposure category. To do this, we identified a measure of central tendency for each category of exposure. Under the assumption that PFOS has an approximately lognormal distribution, three quantiles were chosen to identify the parameters of a lognormal distribution that best matches the reported statistics of the exposure distribution for each study. Depending on how the original authors characterized their exposure distribution, the median and either the 25^th^ and 75^th^ percentiles or the 33^rd^ and 66^th^ percentiles were used. In this study, the function “get.lnorm.par” was used from the R library rriskDistributions for this conversion. Once the parameters of the distribution were identified, we estimated the exposure contrast by calculating the average value of PFOS for each category of exposure (truncated moment). The exposure contrast for each non-referent category was defined as the difference in average PFOS concentration between the non-referent category and the referent category.

Step 2 was used to take the reported beta for each nonreferent exposure category and divide it by the corresponding exposure contrast. For example, the β (difference in g birth weight) for the second tertile of PFOS as compared with the first would be divided by the ng/mL difference in mean PFOS between the second and first tertile, and a similar calculation would be done for the third tertile as compared with the first tertile. The confidence limits were similarly re-scaled.

Step 3 was used to calculate the inverse-variance weighted average of the rescaled beta coefficients across non-referent categories of exposure, where the weight (w) for each coefficient = 1/SE^2^, and the standard error, SE, for each coefficient = (CI_U_ - CI_L_)/3.92. Note that the SE here is based on the re-scaled confidence limits calculated in Step 2. The standard error for the summary β, SE(β), was 1/√Σw.

Because this method combines the standard error from several categorical estimates, we had originally anticipated that the summary β values re-expressed in this manner would overstate the precision of β and require adjustment of the estimated error. However, when we applied this method to five studies that reported both continuous and categorical analyses, the standard error for the published continuous estimates were smaller than the standard error for the approximated continuous estimates in most cases (see Suppl Table 7). As the relationship between the published and estimated standard error was inconsistent and because the typical finding of re-expressed effect estimates being more uncertain may capture some of the uncertainty originating from the categorical to continuous re-expression, we chose not to adjust the estimated standard error for the studies that reported only categorical analysis.

## Supplemental Table 7. Comparison of continuous effect estimates (and confidence intervals) that were reported with values estimated from categorical analyses reported by the authors. β values are in units of g/ng/mL unless otherwise noted.

| **Study** | **Reported Effect Estimate (β (95%CI))** | **Estimated Effect Estimate (β (95%CI))** | **SE Ratio (Published / Estimated)** |
| --- | --- | --- | --- |
| Shoaff et al., 2018^a^ | -0.06 (-0.16, 0.04) | -0.08 (-0.22, 0.07) | 0.69 |
| Wikstrom et al., 2019 | -46.00 (-88.00, -3.00) | -49.50 (-105.08, 6.08) | 0.76 |
| Bach et al., 2016^b^ | -2.92 (-8.33, 2.29) | -11.98 (-20.97, -2.99) | 0.59 |
| Sagiv et al., 2018^c^ | -1.12 (-2.56, 0.32) | -4.34 (-9.50, 0.81) | 0.28 |
| Starling et al., 2017 | -13.80 (-53.80, 26.30) | -18.19 (-34.82, -1.55) | 2.41 |

Abbreviations: SE = standard error; IQR = interquartile range

^a^β values in units of z-score/ng/mL.

^b^The published continuous value was converted from g/IQR to g/ng/mL by dividing by the IQR (4.8 ng/mL).

^c^The published continuous value was converted from g/IQR to g/ng/mL by dividing by the IQR (16.0 ng/mL).

Six studies reported BW changes based on categorical exposures only [10, 23, 25, 35, 42, 57]. For Gardener et al. [10], the results are presented as quartile-specific birthweight Z score. The re-expression procedure in this case was modified to account for this. First, it was necessary to scan the data to get numerical values for the quartile-specific Z-scores and confidence interval. Then, we fit a weighted least-squares model (with intercept) of birthweight Z score as a function of PFOS. The independent variable (PFOS) was the quartile-specific mean PFOS concentration. The model provided a β with units Z-score per ng/mL of PFOS. As a final step, this coefficient was re-expressed as g birth weight/ng/mL PFOS by using the mean and standard deviation of birth weight in their Table 1. The standard error for the re-expressed value was derived from the p-value that Gardener et al. study reported in their Figure 5.

## **Supplemental Figure 1.** Enhanced Funnel Plot of 53 studies included, plus 4 studies imputed, examining PFOS exposures and birth weight differences.


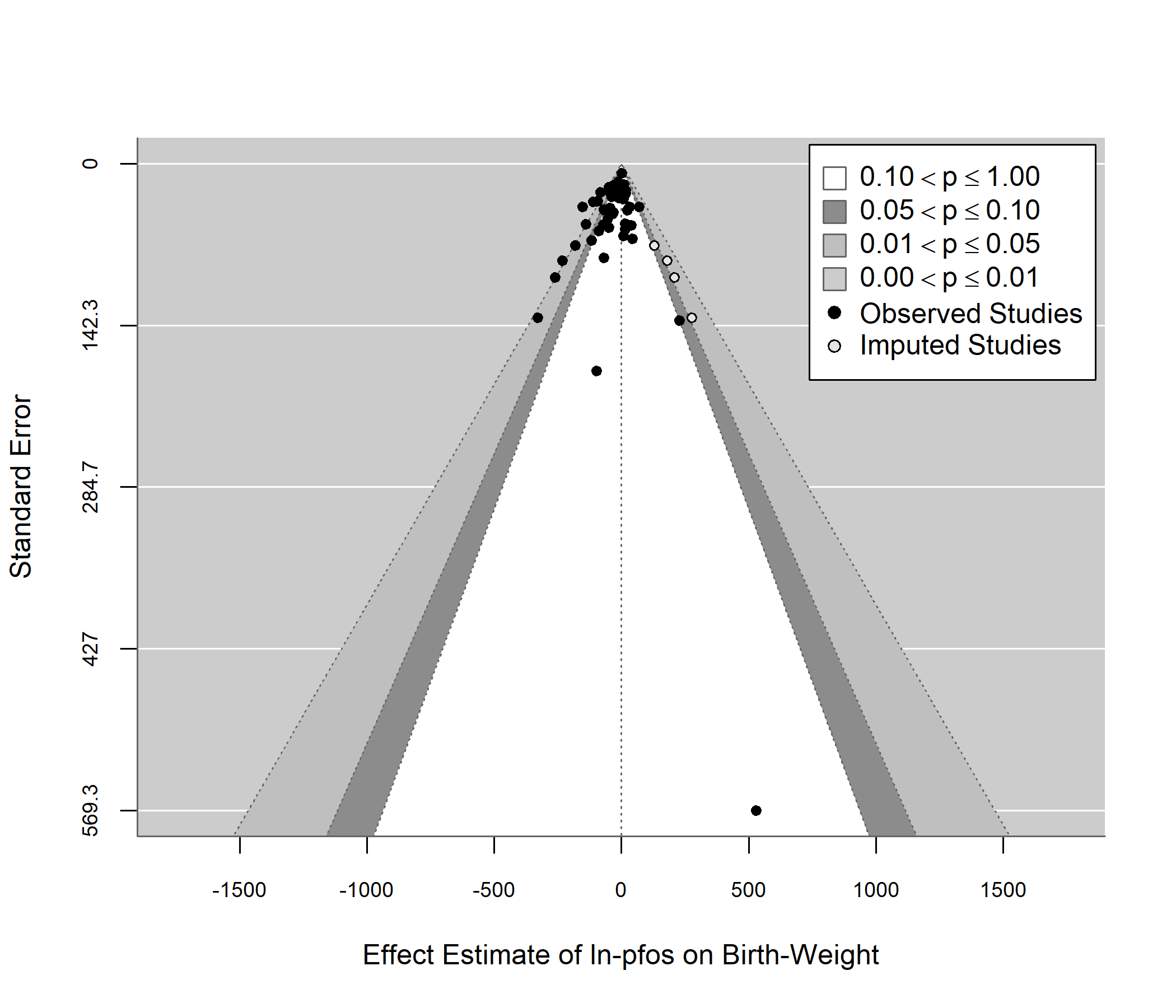


p-value for the test for Funnel plot asymmetry p=0.81

***REFERENCES***

1. Jarvis AL, Justice JR, Elias MC, Schnitker B, Gallagher K. Perfluorooctane Sulfonate in US Ambient Surface Waters: A Review of Occurrence in Aquatic Environments and Comparison to Global Concentrations. Environ Toxicol Chem. 2021 Sep;40(9):2425-2442. doi: 10.1002/etc.5147. Epub 2021 Aug 13. PMID: 34187091; PMCID: PMC9327793.
2. U.S. EPA 2024a. Office of Water Final Human Health Toxicity Assessment for Perfluorooctane Sulfonic Acid (PFOS). 815-R-24-007. April 2024.
3. Dzierlenga MW, Crawford L, Longnecker MP. Birth weight and perfluorooctane sulfonic acid: a random-effects meta-regression analysis. Environ Epidemiol. 2020 Apr 23;4(3):e095.
4. U.S. EPA, 2022. ORD Staff Handbook for Developing IRIS Assessments. U.S. EPA Office of Research and Development, Washington, DC. EPA/600/R-22/268. <https://cfpub.epa.gov/ncea/iris_drafts/recordisplay.cfm?deid=356370>.
5. U.S. EPA, 2021. Systematic Review Protocol for the PFAS IRIS Assessments. U.S. Environmental Protection Agency, Washington, DC. EPA/635/R-19/050. <http://cfpub.epa.gov/ncea/iris_drafts/recordisplay.cfm?deid=345065>.
6. Moher D, Liberati A, Tetzlaff J, Altman DG; PRISMA Group. Preferred reporting items for systematic reviews and meta-analyses: the PRISMA statement. Int J Surg. 2010;8(5):336-41. doi: 10.1016/j.ijsu.2010.02.007. Epub 2010 Feb 18. Erratum in: Int J Surg. 2010;8(8):658. PMID: 20171303.
7. U.S. EPA 2024b. Appendix—Office of Water Final Human Health Toxicity Assessment for Perfluorooctane Sulfonic Acid (PFOS). 815–R–24–009. <https://www.epa.gov/system/files/documents/2024-05/appendix-final-human-health-toxicity-assessment-pfos.pdf>.
8. U.S. EPA, 2016. Drinking water health advisory for perfluorooctane sulfonate (PFOS). 822-R-16-004. May 2016.
9. U.S. EPA. 2024c. Toxicological Review of Perfluorodecanoic Acid (PFDA) and Related Salts (Final Report, 2024). U.S. Environmental Protection Agency, Washington, DC, EPA/635/R-24/172Fa, 2024.
10. Gardener H, Sun Q, Grandjean P. PFAS concentration during pregnancy in relation to cardiometabolic health and birth outcomes. Environ Res. 2021 Jan;192:110287. doi: 10.1016/j.envres.2020.110287. Epub 2020 Oct 8. PMID: 33038367; PMCID: PMC7736328.
11. Yao Q, Gao Y, Zhang Y, Qin K, Liew Z, Tian Y. Associations of paternal and maternal per- and polyfluoroalkyl substances exposure with cord serum reproductive hormones, placental steroidogenic enzyme and birth weight. Chemosphere 2021 Dec;285:131521.
12. Rohatgi A. WebPlotDigitizer version 4.6, Pacifica, CA, USA. https://plotdigitizer.com/. 2022.
13. Ehresman DJ, Froehlich JW, Olsen GW, Chang SC, Butenhoff JL. Comparison of human whole blood, plasma, and serum matrices for the determination of perfluorooctanesulfonate (PFOS), perfluorooctanoate (PFOA), and other fluorochemicals. Environ Res. 2007 Feb;103(2):176-84.
14. Barr DB, Kannan K, Cui Y, Merrill L, Petrick LM, Meeker JD, et al. The use of dried blood spots for characterizing children's exposure to organic environmental chemicals. Environ Res. 2021 Apr;195:110796.
15. Ru H, Lee AL, Rappazzo K, Dzierlenga MW, Radke E, Bateson TF, et al. Systematic Review and Meta-analysis of Birth Weight and Perfluorohexane Sulfonate Exposures: Examination of Sample Timing and Study Confidence. Occupational and Environmental Medicine. 2024;81(5):266-276. doi: 10.1136/oemed-2023-109328.
16. Wright JM, Lee AL, Rappazzo K, Ru H, Radke E, Bateson TF. Systematic Review and Meta-analysis of Birth Weight and PFNA Exposures. Environmental Research 2023, April 1:222:115357. doi: 10.1016/j.envres.2023.115357. Epub 2023 Jan 24. PMID: 36706898.
17. Steenland K, Barry V, Savitz D. Serum perfluorooctanoic acid and birthweight: an updated meta-analysis with bias analysis. Epidemiology 2018;29:765–776.
18. Higgins JPT, Thomas J, Chandler J, Cumpston M, Li T, Page MJ, et al (editors). Cochrane Handbook for Systematic Reviews of Interventions version 6.4 (updated August 2023). Cochrane, 2023. Available from [www.training.cochrane.org/handbook](http://www.training.cochrane.org/handbook).
19. Borenstein M, Hedges LV, Higgins JPT, Rothstein HR. Publication Bias. In Introduction to Meta-Analysis (eds M. Borenstein, L.V. Hedges, J.P.T. Higgins and H.R. Rothstein). 2009. <https://doi.org/10.1002/9780470743386.ch30>.
20. American College of Obstetrics and Gynecology. How Your Fetus Grows During Pregnancy | ACOG, last accessed 12-3-24.
21. Gui SY, Chen YN, Wu KJ, Liu W, Wang WJ, Liang HR, et al. Association Between Exposure to Per- and Polyfluoroalkyl Substances and Birth Outcomes: A Systematic Review and Meta-Analysis. Front Public Health. 2022 Mar 24;10:855348. doi: 10.3389/fpubh.2022.855348.
22. Callan AC, Rotander A, Thompson K, Heyworth J, Mueller JF, Odland JØ, et al. Maternal exposure to perfluoroalkyl acids measured in whole blood and birth outcomes in offspring. Sci Total Environ. 2016 Nov 1;569-570:1107-1113. doi: 10.1016/j.scitotenv.2016.06.177.
23. Cao W, Liu X, Liu X, Zhou Y, Zhang X, Tian H, et al. Perfluoroalkyl substances in umbilical cord serum and gestational and postnatal growth in a Chinese birth cohort. Environ Int. 2018 Jul;116:197-205. doi: 10.1016/j.envint.2018.04.015. Epub 2018 Apr 23.
24. Espindola Santos AS, Meyer A, Dabkiewicz VE, Câmara VM, Asmus CIRF. Serum levels of perfluorooctanoic acid and perfluorooctane sulfonic acid in pregnant women: Maternal predictors and associations with birth outcomes in the PIPA Project. J Obstet Gynaecol Res. 2021 Sep;47(9):3107-3118. doi: 10.1111/jog.14883. Epub 2021 Jun 10. PMID: 34111903.
25. Gao K, Zhuang T, Liu X, Fu J, Zhang J, Fu J, et al. Prenatal Exposure to Per- and Polyfluoroalkyl Substances (PFASs) and Association between the Placental Transfer Efficiencies and Dissociation Constant of Serum Proteins-PFAS Complexes. Environ Sci Technol. 2019 Jun 4;53(11):6529-6538. doi: 10.1021/acs.est.9b00715. Epub 2019 May 23. PMID: 31099564.
26. Mwapasa M, Huber S, Chakhame BM, Maluwa A, Odland ML, Röllin H, Choko A, et al. Serum Concentrations of Selected Poly- and Perfluoroalkyl Substances (PFASs) in Pregnant Women and Associations with Birth Outcomes. A Cross-Sectional Study from Southern Malawi. Int J Environ Res Public Health. 2023 Jan 17;20(3):1689. doi: 10.3390/ijerph20031689. PMID: 36767049; PMCID: PMC9914481.
27. Shi Y, Yang L, Li J, Lai J, Wang Y, Zhao Y, et al. Occurrence of perfluoroalkyl substances in cord serum and association with growth indicators in newborns from Beijing. Chemosphere 2017;169:396–402. <https://doi.org/10.1016/j.chemosphere.2016.11.050>.
28. Workman CE, Becker AB, Azad MB, Moraes TJ, Mandhane PJ, Turvey SE, et al. Associations between concentrations of perfluoroalkyl substances in human plasma and maternal, infant, and home characteristics in Winnipeg, Canada. Environ Pollut. 2019 Jun;249:758-766. doi: 10.1016/j.envpol.2019.03.054. Epub 2019 Mar 21. PMID: 30933773.
29. Xu C, Yin S, Liu Y, Chen F, Zhong Z, Li F, et al. Prenatal exposure to chlorinated polyfluoroalkyl ether sulfonic acids and perfluoroalkyl acids: potential role of maternal determinants and associations with birth outcomes. J. Hazard Mater 2019;380:120867. <https://doi.org/10.1016/j.jhazmat.2019.120867>.
30. Zhang B, Wei Z, Gu C, Yao Y, Xue J, Zhu H, et al. First Evidence of Prenatal Exposure to Emerging Poly- and Perfluoroalkyl Substances Associated with E-Waste Dismantling: Chemical Structure-Based Placental Transfer and Health Risks. Environ Sci Technol. 2022 Dec 6;56(23):17108-17118. doi: 10.1021/acs.est.2c05925. Epub 2022 Nov 18. PMID: 36399367.
31. Apelberg BJ, Witter FR, Herbstman JB, Calafat AM, Halden RU, Needham LL, et al. Cord serum concentrations of perfluorooctane sulfonate (PFOS) and perfluorooctanoate (PFOA) in relation to weight and size at birth. Environ Health Perspect. 2007 Nov;115(11):1670-6. doi: 10.1289/ehp.10334. PMID: 18008002; PMCID: PMC2072847.
32. Cai D, Li QQ, Mohammed Z, Chou WC, Huang J, Kong M, et al. Fetal Glucocorticoid Mediates the Association between Prenatal Per- and Polyfluoroalkyl Substance Exposure and Neonatal Growth Index: Evidence from a Birth Cohort Study. Environ Sci Technol. 2023 Aug 8;57(31):11420-11429. doi: 10.1021/acs.est.2c08831. Epub 2023 Jul 26. PMID: 37494580.
33. Chang CJ, Barr DB, Ryan PB, Panuwet P, Smarr MM, Liu K, et al. Per- and polyfluoroalkyl substance (PFAS) exposure, maternal metabolomic perturbation, and fetal growth in African American women: A meet-in-the-middle approach. Environ Int. 2022 Jan;158:106964. doi: 10.1016/j.envint.2021.106964. Epub 2021 Nov 1.
34. Chen MH, Ha EH, Wen TW, Su YN, Lien GW, Chen CY, et al. Perfluorinated compounds in umbilical cord blood and adverse birth outcomes. PLoS One. 2012;7(8):e42474. doi: 10.1371/journal.pone.0042474. Epub 2012 Aug 3. PMID: 22879996; PMCID: PMC3411780.
35. de Cock M, De Boer MR, Lamoree M, Legler J, Van De Bor M. Prenatal exposure to endocrine disrupting chemicals and birth weight-A prospective cohort study. J Environ Sci Health A Tox Hazard Subst Environ Eng. 2016 Jan 28;51(2):178-185. doi: 10.1080/10934529.2015.1087753. Epub 2015 Nov 25. PMID: 26605905.
36. Gyllenhammar I, Diderholm B, Gustafsson J, Berger U, Ridefelt P, Benskin JP, et al. Perfluoroalkyl acid levels in first-time mothers in relation to offspring weight gain and growth. Environ Int 2018 Feb; 111:191–199. https://doi.org/10.1016/j.envint.2017.12.002. Epub 2017 Dec 20. PMID:29223808.
37. Hamm MP, Cherry NM, Chan E, Martin JW, Burstyn I. Maternal exposure to perfluorinated acids and fetal growth. J Expo Sci Environ Epidemiol. 2010 Nov;20(7):589-97. doi: 10.1038/jes.2009.57. Epub 2009 Oct 28. PMID: 19865074.
38. Hjermitslev MH, Long M, Wielsøe M, Bonefeld-Jørgensen EC. Persistent organic pollutants in Greenlandic pregnant women and indices of foetal growth: the ACCEPT study. Sci. Total Environ 2020;698:134118.
39. Kashino I, Sasaki S, Okada E, Matsuura H, Goudarzi H, Miyashita C, et al. Prenatal exposure to 11 perfluoroalkyl substances and fetal growth: a large-scale, prospective birth cohort study. Environ. Int 2020;136:105355. <https://doi.org/10.1016/j.envint.2019.105355>.
40. Kwon EJ, Shin JS, Kim BM, Shah-Kulkarni S, Park H, Kho YL, et al. Prenatal exposure to perfluorinated compounds affects birth weight through GSTM1 polymorphism. J. Occup. Environ. Med 2016;58:e198–e205. <https://doi.org/10.1097/JOM.0000000000000739>.
41. Lenters V, Portengen L, Rignell-Hydbom A, Jönsson BA, Lindh CH, Piersma AH, et al. Prenatal Phthalate, Perfluoroalkyl Acid, and Organochlorine Exposures and Term Birth Weight in Three Birth Cohorts: Multi-Pollutant Models Based on Elastic Net Regression. Environ Health Perspect. 2016 Mar;124(3):365-72. doi: 10.1289/ehp.1408933. Epub 2015 Jun 26. PMID: 26115335; PMCID: PMC4786980.
42. Maisonet M, Terrell ML, McGeehin MA, Christensen KY, Holmes A, Calafat AM, et al. Maternal concentrations of polyfluoroalkyl compounds during pregnancy and fetal and postnatal growth in British girls. Environ Health Perspect 2012;120:1432–1437. doi:10.1289/ehp.1003096.
43. Meng Q, Inoue K, Ritz B, Olsen J, Liew Z. Prenatal exposure to perfluoroalkyl substances and birth outcomes; an updated analysis from the Danish National Birth Cohort. J Environ Res Public Health 2018;15(9):1832. doi: 10.3390/ijerph15091832.
44. Peterson AK, Eckel SP, Habre R, Yang T, Faham D, Amin M, et al. Detected prenatal perfluorooctanoic acid (PFOA) exposure is associated with decreased fetal head biometric parameters in participants experiencing higher perceived stress during pregnancy in the MADRES cohort. Environ Adv. 2022 Oct;9:100286. doi: 10.1016/j.envadv.2022.100286. Epub 2022 Sep 8. PMID: 36507367; PMCID: PMC9731234.
45. Robledo CA, Yeung E, Mendola P, Sundaram R, Maisog J, Sweeney AM, et al. Preconception maternal and paternal exposure to persistent organic pollutants and birth size: the LIFE study. Environ Health Perspect. 2015;123:88–94. doi: 10.1289/ehp.1308016.
46. Sevelsted A, Gürdeniz G, Rago D, Pedersen CT, Lasky-Su JA, Checa A, et al. Effect of perfluoroalkyl exposure in pregnancy and infancy on intrauterine and childhood growth and anthropometry. Sub study from COPSAC2010 birth cohort. EBioMedicine. 2022 Sep;83:104236. doi: 10.1016/j.ebiom.2022.104236. Epub 2022 Aug 26. PMID: 36030647; PMCID: PMC9434040.
47. Siwakoti RC, Cathey A, Ferguson KK, Hao W, Cantonwine DE, Mukherjee B, et al. Prenatal per- and polyfluoroalkyl substances (PFAS) exposure in relation to preterm birth subtypes and size-for-gestational age in the LIFECODES cohort 2006-2008. Environ Res. 2023 Nov 15;237(Pt 2):116967. doi: 10.1016/j.envres.2023.116967. Epub 2023 Aug 25. PMID: 37634691; PMCID: PMC10913455.
48. Wang H, Du H, Yang J, Jiang H, O K, Xu L, et al. PFOS, PFOA, estrogen homeostasis, and birth size in Chinese infants. Chemosphere. 2019 Apr;221:349-355. doi: 10.1016/j.chemosphere.2019.01.061. Epub 2019 Jan 9. PMID: 30641376.
49. Wang Z, Zhang J, Dai Y, Zhang L, Guo J, Xu S, et al. Mediating effect of endocrine hormones on association between per- and polyfluoroalkyl substances exposure and birth size: Findings from Sheyang Mini Birth Cohort Study. Environ Res. 2023a;226:115658. doi: 10.1016/j.envres.2023.115658. Epub 2023 Mar 8. PMID: 36894112.
50. Zheng T, Kelsey K, Zhu C, Pennell KD, Yao Q, Manz KE, et al. Adverse birth outcomes related to concentrations of per- and polyfluoroalkyl substances (PFAS) in maternal blood collected from pregnant women in 1960-1966. Environ Res. 2024 Jan 15;241:117010. doi: 10.1016/j.envres.2023.117010. Epub 2023 Sep 9. PMID: 37696323.
51. Ashley-Martin J, Dodds L, Arbuckle TE, Bouchard MF, Fisher M, Morriset AS, et al. Maternal Concentrations of Perfluoroalkyl Substances and Fetal Markers of Metabolic Function and Birth Weight. Am J Epidemiol. 2017 Feb 1;185(3):185-193. doi: 10.1093/aje/kww213. PMID: 28172036; PMCID: PMC5391709.
52. Bach CC, Bech BH, Nohr EA, Olsen J, Matthiesen NB, Bonefeld-Jørgensen EC, et al. Perfluoroalkyl acids in maternal serum and indices of fetal growth: the Aarhus Birth Cohort. Environ Health Perspect 2016; 124:848–854. https://doi.org/ 10.1289/ehp.1510046.
53. Bell EM, Yeung EH, Ma W, Kannan K, Sundaram R, Smarr MM, et al. Concentrations of endocrine disrupting chemicals in newborn blood spots and infant outcomes in the upstate KIDS study. Environ Int. 2018 Dec;121(Pt 1):232-239. doi: 10.1016/j.envint.2018.09.005. Epub 2018 Sep 13. PMID: 30219610; PMCID: PMC6376484.
54. Buck Louis GM, Zhai S, Smarr MM, Grewal J, Zhang C, Grantz KL, et al. Endocrine disruptors and neonatal anthropometry, NICHD Fetal Growth Studies - Singletons. Environ Int. 2018 Oct;119:515-526. doi: 10.1016/j.envint.2018.07.024. Epub 2018 Jul 26.
55. Chu C, Zhou Y, Li QQ, Bloom MS, Lin S, Yu YJ, et al. Are perfluorooctane sulfonate alternatives safer? New insights from a birth cohort study. Environ Int. 2020 Feb;135:105365. doi: 10.1016/j.envint.2019.105365. Epub 2019 Dec 9. PMID: 31830731.
56. Darrow LA, Stein CR, Steenland K. Serum perfluorooctanoic acid and perfluorooctane sulfonate concentrations in relation to birth outcomes in the Mid-Ohio Valley, 2005-2010. Environ Health Perspect. 2013 Oct;121(10):1207-13. doi: 10.1289/ehp.1206372. Epub 2013 Jul 9. PMID: 23838280; PMCID: PMC3801459.
57. Eick SM, Hom Thepaksorn EK, Izano MA, Cushing LJ, Wang Y, Smith SC, et alR. Associations between prenatal maternal exposure to per- and polyfluoroalkyl substances (PFAS) and polybrominated diphenyl ethers (PBDEs) and birth outcomes among pregnant women in San Francisco. Environ Health. 2020 Sep 16;19(1):100. doi: 10.1186/s12940-020-00654-2.
58. Govarts E, Remy S, Bruckers L, Den Hond E, Sioen I, Nelen V, et al. Combined Effects of Prenatal Exposures to Environmental Chemicals on Birth Weight. Int J Environ Res Public Health. 2016 May 12;13(5):495. doi: 10.3390/ijerph13050495. PMID: 27187434; PMCID: PMC4881120.
59. Lauritzen HB, Larose TL, Øien T, Sandanger TM, Odland JØ, van de Bor M, et al. Maternal serum levels of perfluoroalkyl substances and organochlorines and indices of fetal growth: a Scandinavian case-cohort study. Pediatr Res. 2017 Jan;81(1-1):33-42. doi: 10.1038/pr.2016.187. Epub 2016 Sep 22. PMID: 27656770; PMCID: PMC5313514.
60. Lind DV, Priskorn L, Lassen TH, Nielsen F, Kyhl HB, Kristensen DM, et al. Prenatal exposure to perfluoroalkyl substances and anogenital distance at 3 months of age in a Danish mother-child cohort. Reprod. Toxicol 2017;68:200–206. <https://doi.org/10.1016/j.reprotox.2016.08.019>.
61. Luo D, Wu W, Pan Y, Du B, Shen M, Zeng L. Associations of prenatal exposure to per- and polyfluoroalkyl substances with the neonatal birth size and hormones in the growth hormone/insulin-like growth factor axis. Environ. Sci. Technol. 2021;55:11859–11873. <https://doi.org/10.1021/acs.est.1c02670>.
62. Manzano-Salgado CB, Casas M, Lopez-Espinosa MJ, Ballester F, Iñiguez C, Martinez D, et al. Prenatal exposure to perfluoroalkyl substances and birth outcomes in a Spanish birth cohort. Environment International 2017;108:278–284.
63. Sagiv SK, Rifas-Shiman SL, Fleisch AF, Webster TF, Calafat AM, Ye X, et al. Early-pregnancy plasma concentrations of perfluoroalkyl substances and birth outcomes in Project Viva: confounded by pregnancy hemodynamics? Am J Epidemiol. 2018;187:793–802. doi: 10.1093/aje/kwx332
64. Shen C, Ding J, Xu C, Zhang L, Liu S, Tian Y. Perfluoroalkyl Mixture Exposure in Relation to Fetal Growth: Potential Roles of Maternal Characteristics and Associations with Birth Outcomes. Toxics. 2022 Oct 28;10(11):650. doi: 10.3390/toxics10110650. PMID: 36355941; PMCID: PMC9695392.
65. Shoaff J, Papandonatos GD, Calafat A.M, Chen A, Lanphear BP, Ehrlich S, et al. Prenatal exposure to perfluoroalkyl substances: infant birth weight and early life growth. Environ Epidemiol 2018;2(2):e10. <https://doi.org/10.1097/EE9.0000000000000010>.
66. Starling AP, Adgate JL, Hamman RF, Kechris K, Calafat AM, Ye X, et al. Perfluoroalkyl substances during pregnancy and offspring weight and adiposity at birth: examining mediation by maternal fasting glucose in the Healthy Start Study. Environ. Health Perspect. 2017;125:067016 https://doi.org/10.1289/EHP641.
67. Valvi D, Oulhote Y, Weihe P, Dalgård C, Bjerve KS, Steuerwald U, et al. Gestational diabetes and offspring birth size at elevated environmental pollutant exposures. Environ. Int. 2017;107:205–215. <https://doi.org/10.1016/j.envint.2017.07.016>.
68. Wang Z, Luo J, Zhang Y, Li J, Zhang J, Tian Y, et al. High maternal glucose exacerbates the association between prenatal per- and polyfluoroalkyl substance exposure and reduced birth weight. Sci Total Environ. 2023b;858(Pt 3):160130. doi: 10.1016/j.scitotenv.2022.160130. Epub 2022 Nov 11. PMID: 36372179.
69. Whitworth KW, Haug LS, Baird DD, Becher G, Hoppin JA, Skjaerven R, et al. Perfluorinated compounds in relation to birth weight in the Norwegian Mother and Child Cohort Study. Am J Epidemiol. 2012 Jun 15;175(12):1209-16. doi: 10.1093/aje/kwr459. Epub 2012 Apr 19. PMID: 22517810; PMCID: PMC3372312.
70. Wikström S, Lin PI, Lindh CH, Shu H, Bornehag CG. Maternal serum levels of perfluoroalkyl substances in early pregnancy and offspring birth weight. Pediatr. Res. 2020;87:1093–1099. https://doi.org/10.1038/s41390-019-0720-1.
71. Xiao C, Grandjean P, Valvi D, Nielsen F, Jensen TK, Weihe P, et al. Associations of Exposure to Perfluoroalkyl Substances With Thyroid Hormone Concentrations and Birth Size. J Clin Endocrinol Metab. 2020 Mar 1;105(3):735–45. doi: 10.1210/clinem/dgz147. PMID: 31665456; PMCID: PMC7112969.
72. Zhang Y, Mustieles V, Martin L, Sun Y, Hillcoat A, Fang X, et al. Maternal and Paternal Preconception Serum Concentrations of Per and Polyfluoroalkyl Substances in Relation to Birth Outcomes. Environ Sci Technol. 2024 Feb 13;58(6):2683-2692. doi: 10.1021/acs.est.3c07954. Epub 2024 Jan 30. PMID: 38290209; PMCID: PMC10924800.
73. Linakis MW, Van Landingham C, Gasparini A, Longnecker MP. Re-expressing coefficients from regression models for inclusion in a meta-analysis. BMC Med Res Methodol 2024;24(1):6. https://doi.org/10.1186/s12874-023-02132-y.
74. Kopylev L, Dzierlenga MW. 2025. The importance of considering variability in re-expression of effect estimate. In Review.
75. Padula AM, Ning X, Bakre S, Barrett ES, Bastain T, Bennett DH, et al. Birth Outcomes in Relation to Prenatal Exposure to Per- and Polyfluoroalkyl Substances and Stress in the Environmental Influences on Child Health Outcomes (ECHO) Program. Environ Health Perspect. 2023 Mar;131(3):37006.
76. Guo P, Warren JL, Deziel NC, Liew Z. Exposure range matters: considering non-linear associations in the meta-analysis of environmental pollutant exposure using examples of per- and polyfluoroalkyl substances and birth outcomes. Am J Epidemiol. 2024 Sep 3:kwae309. doi: 10.1093/aje/kwae309. Epub ahead of print. PMID: 39227151
77. Howard BE, Phillips J, Miller K, Tandon A, Mav D, Shah MR, et al. SWIFT-Review: a text-mining workbench for systematic review. Syst Rev 2016;5: 87.
78. Bjerregaard-Olesen C, Bach CC, Long M, Wielsøe M, Bech BH, Henriksen TB, et al. Associations of Fetal Growth Outcomes with Measures of the Combined Xenoestrogenic Activity of Maternal Serum Perfluorinated Alkyl Acids in Danish Pregnant Women. Environ Health Perspect. 2019 Jan;127(1):17006. doi: 10.1289/EHP1884. PMID: 30676078; PMCID: PMC6381822.
79. Taibl KR, Liang D, Dunlop AL, Barr DB, Smith MR, Steenland K, et al. Pregnancy-related hemodynamic biomarkers in relation to trimester-specific maternal per - and polyfluoroalkyl substances exposures and adverse birth outcomes. Environ Pollut. 2023 Apr 15;323:121331. doi: 10.1016/j.envpol.2023.121331. Epub 2023 Feb 20. PMID: 36813097; PMCID: PMC10023492.
80. Li M, Zeng XW, Qian ZM, Vaughn MG, Sauvé S, Paul G, et al. Isomers of perfluorooctanesulfonate (PFOS) in cord serum and birth outcomes in China: Guangzhou Birth Cohort Study. Environ Int. 2017 May;102:1-8. doi: 10.1016/j.envint.2017.03.006. Epub 2017 Mar 12. PMID: 28297681.
81. Tian Y, Zhou Q, Zhang L, Li W, Yin S, Li F, et al. In utero exposure to per-/polyfluoroalkyl substances (PFASs): Preeclampsia in pregnancy and low birth weight for neonates. Chemosphere. 2023 Feb;313:137490. doi: 10.1016/j.chemosphere.2022.137490. Epub 2022 Dec 10. PMID: 36513200.
82. Shoaff J. October 9, 2018. Personal communication.
83. Woods MM, Lanphear BP, Braun JM, McCandless LC. Gestational exposure to endocrine disrupting chemicals in relation to infant birth weight: a Bayesian analysis of the HOME Study. Environ Health. 2017 Oct 27;16(1):115. doi: 10.1186/s12940-017-0332-3. PMID: 29078782; PMCID: PMC5658906.
84. Kishi R, Nakajima T, Goudarzi H, Kobayashi S, Sasaki S, Okada E, et al. The Association of Prenatal Exposure to Perfluorinated Chemicals with Maternal Essential and Long-Chain Polyunsaturated Fatty Acids during Pregnancy and the Birth Weight of Their Offspring: The Hokkaido Study. Environ Health Perspect. 2015 Oct;123(10):1038-45. doi: 10.1289/ehp.1408834. Epub 2015 Apr 3. PMID: 25840032; PMCID: PMC4590753.
85. Kobayashi S, Azumi K, Goudarzi H, Araki A, Miyashita C, Kobayashi S, et al. Effects of prenatal perfluoroalkyl acid exposure on cord blood IGF2/H19 methylation and ponderal index: The Hokkaido Study. J Expo Sci Environ Epidemiol. 2017 May;27(3):251-259. doi: 10.1038/jes.2016.50. Epub 2016 Aug 24. PMID: 27553991.
86. Minatoya M, Itoh S, Miyashita C, Araki A, Sasaki S, Miura R, et al. Association of prenatal exposure to perfluoroalkyl substances with cord blood adipokines and birth size: The Hokkaido Study on environment and children's health. Environ Res. 2017 Jul;156:175-182. doi: 10.1016/j.envres.2017.03.033. Epub 2017 Mar 27. PMID: 28349882.
87. Swedish Environmental Protection Agency. Rapport till Naturvårdsverkets hälsorelaterade miljöövervakning: Utvärdering av samband mellan mammors POP-belastning under graviditets- och amningsperioden och deras barns hälsa. Report Number 2215-17-008. 2017.
88. Gennings C, Wolk A, Hakansson N, Lindh C, Bornehag CG. Contrasting prenatal nutrition and environmental exposures in association with birth weight and cognitive function in children at 7 years. BMJ Nutr Prev Health. 2020 Jul 26;3(2):162-171. doi: 10.1136/bmjnph-2020-000099. PMID: 33521525; PMCID: PMC7841844.
89. Yeung EH, Bell EM, Sundaram R, Ghassabian A, Ma W, Kannan K, et al. Examining Endocrine Disruptors Measured in Newborn Dried Blood Spots and Early Childhood Growth in a Prospective Cohort. Obesity (Silver Spring). 2019 Jan;27(1):145-151. doi: 10.1002/oby.22332. PMID: 30569634; PMCID: PMC6309795.
90. Gross RS, Ghassabian A, Vandyousefi S, Messito MJ, Gao C, Kannan K, et al. Persistent organic pollutants exposure in newborn dried blood spots and infant weight status: A case-control study of low-income Hispanic mother-infant pairs. Environ Pollut. 2020 Dec;267:115427. doi: 10.1016/j.envpol.2020.115427. Epub 2020 Aug 15. PMID: 33254620; PMCID: PMC7708683.
91. Marks KJ, Cutler AJ, Jeddy Z, Northstone K, Kato K, Hartman TJ. Maternal serum concentrations of perfluoroalkyl substances and birth size in British boys. Int. J. Hyg Environ. Health 2019;222:889–895. <https://doi.org/10.1016/j.ijheh.2019.03.008>.
92. Chowdhury SF, Prout N, Rivera-Núñez Z, Barrett E, Brunner J, Duberstein Z, et al. PFAS alters placental arterial vasculature in term human placentae: A prospective pregnancy cohort study. Placenta. 2024 Apr;149:54-63. doi: 10.1016/j.placenta.2024.03.002. Epub 2024 Mar 9. PMID: 38518389; PMCID: PMC10997442.
93. Lee ES, Han S, Oh JE. Association between perfluorinated compound concentrations in cord serum and birth weight using multiple regression models. Reprod. Toxicol 2016;59:53–59. <https://doi.org/10.1016/j.reprotox.2015.10.020>.
94. Monroy R, Morrison K, Teo K, Atkinson S, Kubwabo C, Stewart B, et al. Serum levels of perfluoroalkyl compounds in human maternal and umbilical cord blood samples. Environ Res. 2008 Sep;108(1):56-62. doi: 10.1016/j.envres.2008.06.001. Epub 2008 Jul 22. PMID: 18649879.
